# Finding Hybrid Incompatibilities Using Genome Sequences from Hybrid Populations

**DOI:** 10.1093/molbev/msab168

**Published:** 2021-06-07

**Authors:** Alexandre Blanckaert, Bret A Payseur

**Affiliations:** Laboratory of Genetics, University of Wisconsin-Madison, Madison, WI, USA

**Keywords:** genetic incompatibilities, hybrid zone, site frequency spectrum, epistasis

## Abstract

Natural hybrid zones offer a powerful framework for understanding the genetic basis of speciation in progress because ongoing hybridization continually creates unfavorable gene combinations. Evidence indicates that postzygotic reproductive isolation is often caused by epistatic interactions between mutations in different genes that evolved independently of one another (hybrid incompatibilities). We examined the potential to detect epistatic selection against incompatibilities from genome sequence data using the site frequency spectrum (SFS) of polymorphisms by conducting individual-based simulations in SLiM. We found that the genome-wide SFS in hybrid populations assumes a diagnostic shape, with the continual input of fixed differences between source populations via migration inducing a mass at intermediate allele frequency. Epistatic selection locally distorts the SFS as non-incompatibility alleles rise in frequency in a manner analogous to a selective sweep. Building on these results, we present a statistical method to identify genomic regions containing incompatibility loci that locates departures in the local SFS compared with the genome-wide SFS. Cross-validation studies demonstrate that our method detects recessive and codominant incompatibilities across a range of scenarios varying in the strength of epistatic selection, migration rate, and hybrid zone age. Our approach takes advantage of whole genome sequence data, does not require knowledge of demographic history, and can be applied to any pair of nascent species that forms a hybrid zone.

## Introduction

Under the biological species concept, species formation results from the accumulation of reproductive barriers between lineages (Dobzhansky 1937; Mayr 1942). Reproductive isolation can be mediated by the environment (extrinsic isolation) or by genetic factors (intrinsic isolation). Modes of intrinsic isolation can be broadly categorized as involving processes that precede zygote formation (prezygotic) or involving processes that affect the development of the organism (postzygotic).

A popular explanation for postzygotic isolation is the Bateson−Dobzhansky−Muller (BDM) model (Bateson 1909; Dobzhansky 1936; Muller 1942). The model postulates that reproductive isolation arises when geographically separate populations fix incompatible mutations in genes that normally interact. Hybrids carrying incompatible combinations (referred to as Dobzhansky−Muller incompatibilities or hybrid incompatibilities) suffer reduced viability, reduced fertility, or both. Theoretical examination of the BDM model has produced important insights into speciation, including the extent to which incompatibilities inhibit gene flow between populations (Bank et al. 2012; Blanckaert and Hermisson 2018; Blanckaert et al. 2020) and the rate at which incompatibilities accumulate over time (Orr 1995; Turelli and Orr 2000; Orr and Turelli 2001).

The genes that form hybrid incompatibilities can provide glimpses into the genetic mechanisms responsible for postzygotic isolation (Presgraves 2010; Maheshwari and Barbash 2011). An incompatibility between two genes required to repress transcription of transposable elements and satellite DNA (Satyaki et al. 2014), *Hmr* and *Lhr*, kills hybrids between *Drosophila simulans* and *D. melanogaster* (Brideau et al. 2006). Dysfunctional interactions between *Xmrk* and (likely) *Rab3d* cause lethal melanomas in hybrids between *Xiphophorus maculatus* and *X. hellerii* (Lu et al. 2020). *Xmrk* also forms a potential incompatibility with a different gene, *Cd97*, in a cross between two other species of swordtail fish (*X. malinche* and *X. brichmanni*) (Powell et al. 2020). Two genes, *Sgs1* and *Msh2*, have recently been shown to largely contribute to the genetic barrier between *Saccharomyces cerevisiae* and *S. paradoxus* (Bozdag et al. 2021), by preventing the formation of crossovers between diverged sequences. Finally, *Prmd9*, a gene that regulates recombination hotspots, causes male sterility in hybrid mice (Mihola et al. 2009).

Although mapping and characterization of hybrid incompatibilities in the laboratory remains the best way to discover mechanisms of postzygotic isolation, the necessary experiments require substantial time. This framework also focuses on single hybrid phenotypes that are visible and relatively simple, whereas nascent species are often separated by a multitude of complex reproductive barriers. In one example, Martin and Wainwright (2013) demonstrated the existence of postzygotic isolation in *Cyprinodon* pupfishes and identified phenotypes affected by incompatibilities. However, given the complexity of the phenotypes, as well as the extrinsic nature of the interactions, the underlying genetic interactions were not investigated.

The examination of patterns of gene flow in natural hybrid populations is a compelling alternative to genetic dissection of reproductive barrier phenotypes in the lab. This framework features the advantages of focusing on fitness effects of hybridization in the wild, applying to many nascent species pairs that cannot be crossed in captivity, avoiding assumptions about the phenotypic basis of isolation, and considering hybrid individuals after many generations of admixture. In addition, genes identified using natural hybrid populations are likely involved in speciation, whereas incompatible genes identified between species that no longer naturally hybridize might have diverged after speciation was complete.

Several statistical methods have been developed with the goal of characterizing natural selection against hybrids ( reviewed by Payseur 2010; Payseur and Rieseberg 2016). A strategy based on geographic clines (Porter et al. 1997) builds on theory that equates changes in allele frequency across transects of a hybrid zone to a balance between selection and migration (Barton and Hewitt 1985; Szymura and Barton 1986). A framework based on genomic clines searches for loci that show collections of genotypes that depart from genome-wide hybrid indices (Rieseberg et al. 1999; Gompert and Buerkle 2009, 2011). Building on empirical studies that identified unlinked pairs of loci showing strong associations between conspecific alleles in collections of hybrids (Payseur and Hoekstra 2005; Teeter et al. 2008; Schumer et al. 2014; Pool 2015), another approach looks for loci displaying stronger admixture disequilibrium than the remainder of the genome (Schumer and Brandvain 2016). Collectively, these analytical strategies for identifying loci that experience selection against hybrids face challenges. Although methods that focus on geographic clines or genomic clines have found loci that likely reduce fitness in hybrids, these methods were not designed for application to whole genome sequences. Despite the intuitive appeal of using admixture disequilibrium to detect epistatic selection, simulations suggest this approach is unreliable, even when population structure is accounted for (Schumer and Brandvain 2016). As genomic data from hybrid populations continue to accumulate, new methods that locate hybrid incompatibilities using genome sequences would accelerate progress toward deciphering the genetics of speciation.

A potentially general signature of selection against incompatibilities comes from consideration of the effects of epistatic selection in hybrid populations. When a heterospecific combination of alleles at two loci decreases hybrid fitness, selection reduces the number of individuals with this genotype. As a result, the population frequencies of the compatible alleles increase. Neutral mutations linked to each compatible allele should also spread through the population in a manner partly analogous to a selective sweep (Maynard Smith and Haigh 1974). Therefore, we might expect the site frequency spectrum (SFS) of polymorphisms in genomic regions containing hybrid incompatibilities to depart from the genome-wide SFS (Braverman et al. 1995; Simonsen et al. 1995).

In this paper, we present a method to locate hybrid incompatibilities using the SFS. Following the logic of successful approaches developed to detect selective sweeps in nonhybrid populations (Nielsen 2005; Williamson et al. 2007), we deploy the full SFS (rather than summarizing it) and we use the genomic spectrum as a null model (bypassing the need to specify a demographic model). Simulations of hybrid populations demonstrate that our method finds incompatibilities across a range of scenarios involving epistatic selection, migration, and genetic drift.

### New Approaches

We developed a new method to detect epistatic selection in natural hybrid populations by exploiting information contained in the SFS. We computed the local unfolded SFS along the genome over regions of 500 kb and calculated the probability of observing the local SFS given the global one using a multinomial distribution. Through bootstrapping, we established the distribution of windows of the chromosome that are found within the 1% lower tail of the probability of observing the local SFS given the global SFS. A region was defined as an outlier if it fulfills the two following conditions: The window itself appears often enough in the 1% tail of the distribution and this window and its neighbors appear on average often enough in the 1% tail of the distribution.

## Materials and Methods

### Simulations

We modeled a hybrid zone consisting of one hybrid population and two parental populations, using SLiM v3.4 (Haller and Messer 2019). Initially, the hybrid population was absent. The two parental populations, each of size *N*_p_ = 5,000, began with the same genome except for a fixed difference at each of two loci (derived alleles *A* and *B*), and diverged in allopatry for *n* = 50,000 generations (burn-in phase). New mutations arose at rate $\mu =10^{-9}$ and were assumed to be neutral. After this initial phase, the hybrid population, of size *N* = 10,000, was formed in a single generation as a 50:50 combination of the two parental populations (without depleting them). 

The hybrid population received migrants at rate *m *=* *0.005 from each parental population (for a total of 2m=0.01). There was no migration from the hybrid population to the parental populations. The hybrid population (a sink) formed a barrier to gene flow between the parental populations (sources), which could reflect restrictions on migration imposed by the environment or reduced fitness in hybrids (Barton and Bengtsson 1986). 

Individuals were diploid with genomes composed of one chromosome of length *L* = 500 Mb. Recombination occurred during meiosis at a uniform rate of *r* = 5 × 10^−9^ per base pair. Loci *A* and *B* were situated at positions 175 and 325 Mb, respectively, and their derived alleles interacted epistatically to reduce fitness by ϵ = −0.1. To model incompatibility associated with local adaptation, we also considered fitness disadvantages *s*_a_ and *s*_b_ for the two ancestral alleles (default *s*_a_ = *s*_b_ = 0). Fitness effects were multiplicative, with the fitness of an individual given by

(1) $$w=(1+s_{a}{)}^{2-X_{A}}(1+s_{b}{)}^{2-X_{B}}(1+h_{X_{A}*X_{B}}\epsilon {)}^{X_{A}*X_{B}}.$$

In this equation, *X*_A_ and *X*_B_ indicate the number of derived alleles (0, 1, or 2) at locus *A* and *B*, respectively. Here $h_{X_{A}*X_{B}}$ represents the dominance coefficient for the epistatic interaction. The recessive and codominant cases only differ for the double heterozygote individual *AaBb*: $h_{1}=0$ if the incompatibility is recessive and $h_{1}=1$, if the incompatibility is codominant. For all other cases (k∈0,2,4), *h_k_* = 1 for both recessive and codominant cases. Fitness was equal to the probability that an individual survived to adulthood. All surviving individuals contributed equally to the next generation.

We sampled 50 individuals without replacement from the hybrid population at generations 100, 1,000, 5,000, and 10,000 (with the counter beginning at 0 when the hybrid population was formed). Sampled individuals were not removed from the population.

We used two approaches to make the simulations computationally feasible. We scaled up recombination rates and mutation rates by a factor of 10 to represent a larger chromosome (*Lr* and Lμ were kept constant). In addition, we used a single burn-in in the parental populations for all simulations that assumed the same genomic architecture. The range of parameter values used for simulations is provided in table 1; values given in the paragraphs above were treated as defaults. Our source code is available on zenodo (doi:10.5281/zenodo.4614847).

**Table 1.** Scenarios and Parameter Values Examined.

Parameters			Name Codominant Case	Name Recessive Case
ϵ=-0.1	s=0	m=0.005	Default	Default rec.
ϵ=-0.02	s=0	m=0.005	Low ep.	Low ep. rec
ϵ=-0.5	s=0	m=0.005	High ep.	High ep. rec.
ϵ=-0.1	s=0	m=0.0005	Low mig.	Low mig. rec
ϵ=-0.1	s=0	m=0.05	High mig.	High mig. rec.
ϵ=-0.5	s=0	m=0.05	High mig. high ep.	High mig. high ep. rec.
ϵ=0	s=0	m=0.005	Neu.	NA
ϵ=0	s=-0.02	m=0.005	SL-sel.	NA
ϵ=0	s=-0.1	m=0.005	High SL-sel.	NA
ϵ=-0.1	s=-0.02	m=0.05	SL-sel. and ep.	SL sel. and ep. rec.

### SFS and Outlier Detection

To detect incompatibilities, we computed the unfolded SFS in non-overlapping 500 kb windows (for a total of 1,000 windows in the genome) for samples of 50 diploid individuals. We also tabulated the genome-wide SFS. Single nucleotide polymorphisms (SNPs) with more than two alleles were ignored. For each window, we calculated the probability of observing the local SFS using a multinomial distribution with parameters estimated from the genome-wide SFS. This probability was estimated *n*_b_ = 1,000 times by bootstrap resampling of the 100 sampled sequences. For each bootstrap replicate, we recorded the first percentile of the probability distribution of the local SFS given the genome-wide SFS. Then, we constructed the distribution across bootstrap replicates of the position of the first percentile along the chromosome. We counted the number of times each window appeared in the first percentile of the distribution (*κ*). If *κ* was larger than a threshold value (*thr*_1_, $\kappa >thr_{1}$), the window was identified as a potential outlier. To further reduce false positives, we required that windows adjacent to a potential outlier also appeared in the first percentile, reasoning that the distortion generated by selection was likely to extend beyond 500 kb. Therefore, for each candidate outlier, we calculated *κ_d_*, the average number of times a window in the [i-d,i+d] region was found in the first percentile of the distribution. A candidate outlier was classified as a true outlier if *κ_d_* was larger than a second threshold (*thr*_2_, $\kappa_{d}>thr_{2}$). Given that the initial filter was done on each window independently ($\kappa >thr_{1}$), we considered as false positive any outlier that did not include incompatibility loci *A* or *B* in the [i-d,i+d] region of detection. Therefore, there were 4*d+2 windows that were considered true positives and 1,000-4*d-2 considered false positives. Due to dependence on *d* of the numbers of true positives and false positives, larger values of *d* will perform better. Due to the bootstrap step, there were at most $10,000/thr_{1}$ possible candidate outliers (ranging from 25 if $thr_{1}=400$ to 10 if $thr_{1}=1,000$). Therefore, we used this value ($10,000/thr_{1}$) in Bonferroni corrections for multiple testing.

### Evaluation of Method Performance

For each simulated scenario, we initially calculated the power of the method, the false positive rate and the proportion of false positives among the outliers for combinations of $\left\{thr_{1},thr_{2},d\right\}$ using all available simulations, with *thr*_1_ any value in {400, 450, ..., 1000}, *thr*_2_ any value in {0, 20, ..., 300} and 5≤d≤19. We determined which $\left\{thr_{1},thr_{2},d\right\}$ provided the highest power for each scenario. As mentioned in the previous section, *d* determines the number of true positive regions in the genome, and therefore larger *d* will always have higher power. To compensate for this effect, we also determined the best $\left\{thr_{1},thr_{2},d\right\}$, using two additional optimizing metrics, power/(2*d+1) and $\text{power}/(2*d+1{)}^{2}$ and introducing a penalty for larger values of *d*. If the optimizing metrics were identical, we used resolution (lower is better), then proportion of false positives in outliers (smaller is better), and finally *thr*_2_ (larger is better) to determine the best combination of $\left\{thr_{1},thr_{2},d\right\}$.

Based on these results, we examined further those scenarios for which our method showed at least 50% power, a false positive rate below $5*thr_{1}/10,000$%, and a proportion of false positives among outliers below 5%. For these scenarios, we preserved only combinations of $\left\{thr_{1},thr_{2},d\right\}$ that passed the criteria described above (a false positive rate below $5*thr_{1}/10,000$% and a proportion of false positives among outliers below 5%) and determined the best combination by averaging the optimizing metric over the selected scenarios.

To further evaluate the method, we performed cross-validation. Using a randomly chosen subset of the simulations (25%) from the restricted group described above, we determined which combination $\left\{thr_{1},thr_{2},d\right\}$ provided the best results. We then measured performance on the remaining data set using the values of *thr*_1_, *thr*_2_, and *d* estimated in the previous step, providing cross-validation of our approach. We repeated this step 100 times with power and power/(2*d+1) as optimizing metrics. For each optimizing metric, the mode of the distribution of best $\left\{thr_{1},thr_{2},d\right\}$ was considered to be the best value to detect incompatibilities. In addition to choosing the best combination $\left\{thr_{1},thr_{2},d\right\}$ for each iteration, we also considered keeping any combination of $\left\{thr_{1},thr_{2},d\right\}$ that performed almost as well (with the optimizing metric being within 5% of the best one). This approach allowed us to capture the possible existence of a plateau in the $\left\{thr_{1},thr_{2},d\right\}$ space, therefore obtaining a more robust estimate of an effective $\left\{thr_{1},thr_{2},d\right\}$ combination, at the slight cost of power.

## Results

### Characterization of the SFS in a Hybrid Population

**Fig. 1.**
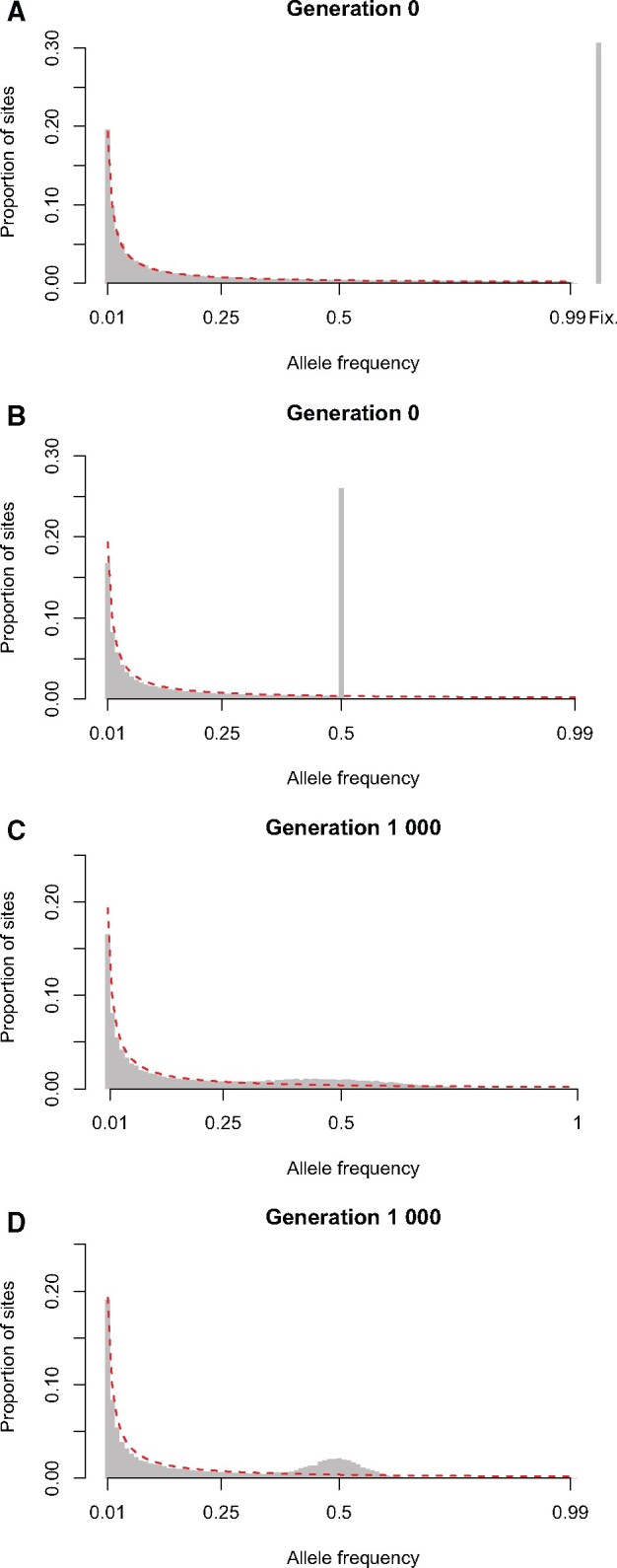
Neutral SFS in a hybrid zone. The neutral expectation of an isolated population is given in red and proportional to 1/*f* (Wright 1938). (*A*) SFS for the parental population at the time of formation of the hybrid population. The relative proportion of fixed mutations (#fixed/#polymorphic) has been added to the right. (*B*) Predicted SFS for the hybrid population at generation 0. (*C*) SFS for an isolated hybrid population at generation 1,000. (*D*) SFS for a sink hybrid population at generation 1,000 (*m *=* *0.005). SFS for both the isolated hybrid populations and the sink hybrid population at different time points (100, 5,000 and 10,000) can be found in supplementary figure S1, Supplementary Material online.

Populations in a hybrid zone display a SFS that departs from what is expected in classical Wright−Fisher populations (where the relative proportion of SNPs with a frequency *f* is proportional to 1/*f* [Wright 1938]), as illustrated in figure 1*A*. Indeed, when initially formed, the hybrid population possesses a unique SFS (fig. 1*B*), characterized by a large proportion of SNPs at frequency 0.5; this proportion depends on the level of divergence between the two parental populations. These SNPs correspond to fixed mutations, private to each parental population (since the hybrid population is formed by a symmetric contribution of the two parental populations). The constant influx of fixed differences between parental populations into the hybrid zone via migration helps to maintain the proportion of SNPs with frequency 0.5, whereas drift spreads the distribution around 0.5 (fig. 1*D*). In the absence of selection, the SFS of a (sink) hybrid population can be decomposed into three components: One part resulting from mutation-drift balance and the initial composition of the hybrid population, and two parts inherited from the parental populations via migration (here, these two parts are indistinguishable due to the symmetric contribution of the parental populations to the hybrid population). In the absence of migration, only the first component remains: The SFS of the initial hybrid population slowly converges toward the SFS of a Wright−Fisher population under drift. After 1,000 generations, the isolated hybrid population still displays an excess of alleles at intermediate frequency (fig. 1*C*), though this effect is far more diffuse than in the presence of migration and almost vanishes after 10,000 generations (supplementary fig. S1, Supplementary Material online). In addition, it is worth noting that although the SFS in the parental populations does not change, the part of the SFS in the hybrid population generated by migration (equivalent to fig. 1*B*) changes as the parental populations continue to diverge and accumulate fixed differences.

**Fig. 2.**
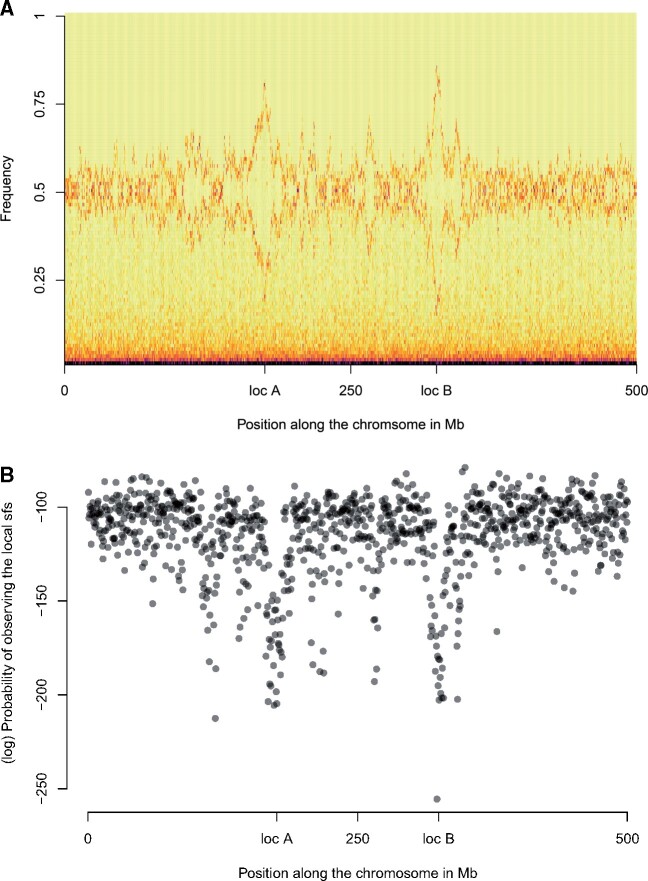
(*A*) Local SFS calculated over regions of 500 kb. The *X*-axis corresponds to the position along the chromosome, the *Y*-axis shows SNP allele frequency. The density is indicated by color, with yellow denoting a lack of SNPs with the corresponding frequency and black denoting an abundance of SNPs with that frequency. (*B*) Probability of observing the local SFS conditional on the global SFS. Results are shown for simulations with s=0,ϵ=-0.1,m=0.005,gen.=1,000.

Epistatic selection against hybrid genotypes shifts the SFS in the vicinity of incompatibility loci (fig. 2*A* for the default scenario; see supplementary figs. S2−S17, Supplementary Material online for other scenarios). The distortion is spatially broad, extending approximately 10 − 20 Mb around incompatibility loci (fig. 2*A*). With epistatic selection against incompatible alleles *A* and *B*, genetic backgrounds containing the ancestral *a* allele or the ancestral *b* allele have a marginal fitness advantage. As a result, derived alleles at neutral SNPs linked to *a* or *b* increase in frequency away from 0.5, creating a detectable, local signature in the SFS. A similar distortion of the SFS is generated by single-locus selection but this distortion is stronger (assuming s=ϵ) because single-locus selection acts independently of the genetic background (supplementary fig. S3, Supplementary Material online) and therefore is always present.

### Finding Genomic Regions with Distortions in the SFS Generated by Epistatic Selection

To quantify the distortion of the SFS near incompatibility loci, we used the multinomial distribution to compute the probability of observing the local SFS (over a 500 kb window) given the global SFS calculated for the entire chromosome. Due to the resulting high dimensionality, the probability of observing a particular SFS was always extremely low (fig. 2*B*). For reference, the probability of observing exactly once each number between 1 and 100 when drawing 100 numbers at random (all having the same probability of being chosen) is 9.33 × 10^−43^. Nevertheless, the probability of observing an SFS near an incompatibility locus is many orders of magnitude lower than the probability of observing one that is similar to the global SFS (fig. 2*B*). Most of the windows with the lowest probabilities of observing the local SFS are found near incompatibility loci, *A* and *B*. However, due to chance, some regions of the chromosome may have a local SFS that differs from the rest of the chromosome (e.g., in fig. 2*B* to the left of locus *A*, around position 125 Mb).

**Fig. 3.**
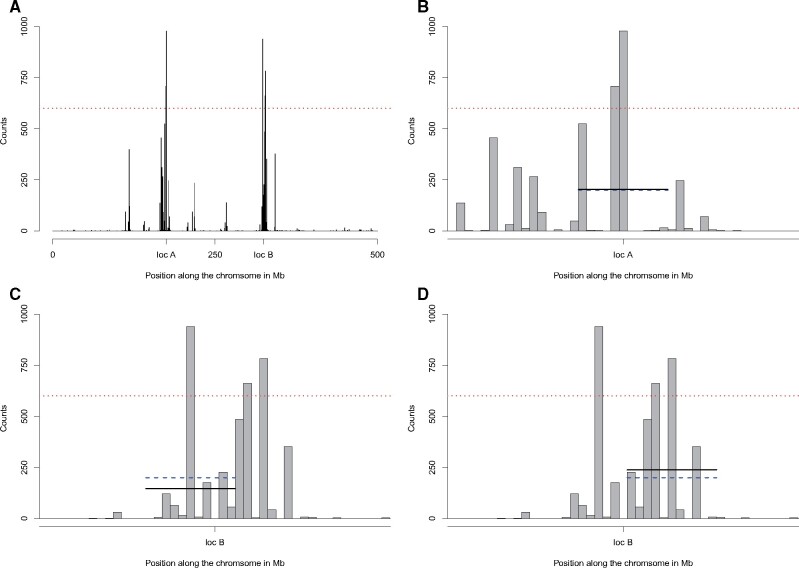
Outcome of bootstrap analysis and definition of thresholds. (*A*) Number of times each window was in the lower 1% tail for the probability of the local SFS being observed given the global SFS in the analysis with 1,000 bootstrap replicates. The red dotted line indicates the first threshold, *thr*_1_. In this example, there are five windows that satisfy this first criterion. (*B*) Zoom-in on the two outlier windows detected close to locus A. The blue dashed line indicates the second threshold *thr*_2_ and the black line indicates the average number of times a window appears in the lower 1% tail. Both the blue line and the black line are drawn only over the windows of interest, that is, including *d *=* *5 windows to the left and five windows to the right of the focal window. (*C*, *D*) Zoom-in on two outlier windows detected close to locus B. The bootstrap analysis depicted in this figure used the same set of data presented in figure 2.

To distinguish SFS signatures due to epistatic selection from those due to chance, we ran a bootstrap analysis to identify outliers. We designated as outliers those regions with probabilities that consistently fell within the lowest 1% of the distribution of probability of observing the local SFS based on the global SFS along the chromosome. Figure 3 illustrates the outcome of the bootstrap analysis for the case of epistatic selection (with ϵ=-0.1). Most of the regions that are detected as outliers more than 50% of the time map near the incompatibility loci (windows 350 and 650; fig. 3*A*). The effects of epistatic selection extend beyond windows containing the incompatibility loci. The bootstrap analysis effectively removes some windows with extreme probabilities while retaining windows adjacent to incompatibility loci. Figure 2 displays an example in which a local SFS in a neutral region around 125 Mb has a very low probability of being observed. After bootstrap analysis, the signal partially vanishes: First this region appears in the 1% tail about 40% of the time; second, the signal is isolated.

The probability of observing a given data set under a multinomial distribution depends on the number of observations as well as how they are partitioned. Just as the binomial probability of observing exactly *n* “heads” with 2*n* coin flips decreases as *n* increases, the multinomial probability of observing a particular SFS decreases as the number of SNPs grows. Although we recover such a relationship between the number of SNPs and the (log) probability of the local SFS, windows with the lowest probabilities are not those with the most SNPs, whether they evolve under neutrality (supplementary fig. S19*A*, Supplementary Material online), single-locus selection (supplementary fig. S19*B*, Supplementary Material online), or epistatic selection (supplementary fig. S19*C* and *D*, Supplementary Material online).

### Method Performance

For each scenario, we calculated the power, false positive rate and proportion of false positives in outliers for our method, considering diverse combinations of $\left\{d,thr_{1},thr_{2}\right\}$. Table 2 shows the combination of $\left\{d,thr_{1},thr_{2}\right\}$ that generates a false positive rate below $5*thr_{1}/10,000$% (Bonferroni correction), a proportion of false positive in outliers below 5%, and the highest power. As mentioned in the Methods, since power increases with the size of the region considered as “true,” 2*d+1, and therefore with *d*, we also considered two additional optimization metrics: 1) power corrected by the number of windows around the true location considered as “true” positive power/(2*d+1) or 2) the square of this value $\text{power}/(2*d+1{)}^{2}$.

**Table 2.** Best Combination of $\left\{d,thr_{1},thr_{2}\right\}$ and Power for the Different Scenarios.

Optimizing Metric:	Power		$\frac{\text{Power}}{2*d+1}$		$\frac{\text{Power}}{(2*d+1{)}^{2}}$	
Scenario	$\left\{d,thr_{1},thr_{2}\right\}$	Power	$\left\{d,thr_{1},thr_{2}\right\}$	Power	$\left\{d,thr_{1},thr_{2}\right\}$	Power
Default	16, 400, 20	0.958	9, 800, 40	0.763	6, 950, 80	0.486
Default rec.	15, 400, 40	0.910	9, 700, 80	0.722	5, 950, 100	0.293
Low epis.	18, 400, 60	0.431	7, 750, 100	0.254	5, 850, 120	0.16
Low epis. rec.	19, 500, 60	0.261	7, 750, 120	0.153	5, 800, 160	0.111
High epis.	18, 500, 20	0.948	9, 950, 60	0.611	9, 950, 60	0.611
High epis. rec.	16, 400, 20	0.986	9, 800, 100	0.770	6, 950, 80	0.481
Low mig.	19, 450, 20	0.915	6, 950, 120	0.669	6, 950, 120	0.669
Low mig. rec.	18, 650, 40	0.836	6, 950, 100	0.579	6, 950, 100	0.579
High mig.	16, 400, 100	0.099	16, 400, 100	0.099	5, 800, 160	0.024
High mig. rec.	16, 400, 100	0.07	5, 800, 100	0.03	5, 800, 100	0.03
High mig. high epis.	16, 400, 100	0.406	16, 400, 100	0.406	5, 950, 100	0.068
High mig. high epis. rec.	18, 400, 80	0.353	18, 400, 80	0.353	5, 900, 120	0.055
SL-sel. and epis.	16, 550, 60	0.548	13, 750, 160	0.466	7, 950, 300	0.154
SL-sel. and epis. rec.	16, 400, 60	0.575	9, 700, 100	0.405	5, 850, 200	0.205

Note.—We consider here three different optimization metrics: power (left columns), power/(2*d+1) (central columns) and $\text{power}/(2*d+1{)}^{2}$ (right columns). Combinations of {$d,thr_{1},thr_{2}$ } leading to a false positive rate above $5*thr_{1}/10,000$% or a proportion of false positive in the outliers above 5% were excluded.

When we ignore the effect of *d* on power, we obtain relatively high power across multiple scenarios, finding the incompatibility loci more than 90% of the time, for both recessive and codominant incompatibilities. The incompatibility loci are found in regions ranging in size from 15.5 Mb (under weak epistatic selection with codominance) to 19.5 Mb (low migration). Reducing the size of the region is possible, at the cost of power. By penalizing for the size of the window of detection, we can narrow the position of the incompatibility loci to less than 10 Mb for multiple scenarios. It can be further narrowed but at a large cost of power. For example, in the default scenario, the window can be reduced by approximately two-thirds to 6.5 Mb with an associated decrease by half in power from 0.958 to 0.486. Therefore, using power/(2*d+1) as the optimization metric offers a good compromise between power and resolution.

When migration is high (10% of the hybrid population is replaced by individuals from the parental sources each generation), we fail to detect the incompatibility loci. In this case, the SFS reflects mainly the genome-wide effects of migration, rather than the balance between migration and selection (supplementary fig. S12, Supplementary Material online). Even when selection against hybrids is extremely strong (F1 hybrid fitness is reduced by 50% in the codominant case, and by 94% for the double homozygote *AABB*), incompatibility loci are detected only 40% of the time and with a rather low resolution (in a 16.5 Mb region). In that case, due to the presence of perpetual strong migration and selection, both incompatible alleles remain at intermediate frequencies (≈0.30 for all 4 time points). There is not enough time for the parental haplotype to fully break down and therefore the distortion of the SFS extends over a rather large region (more than 50 Mb, see supplementary fig. S14, Supplementary Material online), making it a challenge to pinpoint the incompatibilities themselves. When migration is not too strong (1% of the hybrid population is replaced per generation), weakly selected incompatibility loci (ϵ=-0.02) can be potentially detected (≈43% within a 18.5 Mb region or ≈25% within a 7.5 Mb).

When there is both single-locus selection against alleles *a* and *b* and epistatic selection against alleles *A* and *B*, power is reduced by about 40% compared with epistatic selection alone (from 0.958 to 0.548 for the codominant case and from 0.910 to 0.575 for the recessive one, when power is the optimizing metric). This is also true with power/(2*d+1) as the optimization metric (table 2).

Finally, we emphasize that although we obtain a variety of best combinations $\left\{d,thr_{1},thr_{2}\right\}$ for the diverse scenarios, we still find a common combination of $\left\{d,thr_{1},thr_{2}\right\}$ where the method performs close to its best across scenarios.

To further evaluate the method, we performed a cross-validation analysis, using 25% of the simulations (randomly chosen) from scenarios with epistatic selection in which the incompatibility loci were detectable (see Materials and Methods). We report which combination of $\left\{d,thr_{1},thr_{2}\right\}$ was the best for each of the 100 iterations with power/(2*d+1) (table 3) as the optimizing metric (see supplementary table S1, Supplementary Material online for results using power as the optimization metric). The mode of the distribution corresponds to the best combination $\left\{d=9,thr_{1}=900,thr_{2}=60\right\}$, translating into a resolution of 9.5 Mb. The second most common combination, $\left\{d=9,thr_{1}=900,thr_{2}=80\right\}$, is really similar, therefore confirming our result. When we did not penalize for the size of the window *d*, the best combination was observed in 61 out of 100 iterations and given by $\left\{d=16,thr_{1}=400,thr_{2}=40\right\}$ (supplementary table S1, Supplementary Material online).

**Table 3.** Distribution of Combination of $\left\{d,thr_{1},thr_{2}\right\}$ Using (power/(2*d+1)) as the Optimizing Metric.

*d*	*thr*_1_	*thr*_2_	Count (best)	Count (top5%)
9	900	60	43	56
9	900	80	28	81
9	900	40	9	10
9	900	100	8	32
9	850	100	4	5
9	950	40	2	4
9	900	0	0	10
9	900	20	0	10

Note.—Fourth column corresponds to the number of a time a combination is the best one, the fifth one to the number of times a combination is within 5% of the best combination. Only combinations that appeared twice as the best combination, or five times within 5% of the best combination, were displayed.

Since the proportion of false positives in outliers is one of the criteria we consider, the effects of *thr*_1_ and *thr*_2_ are not straightforward. Indeed, although the false positive rate will decrease (or remain unchanged in the worst-case scenario) when the filters become more stringent, the proportion of false positives among outliers may increase, due to the possible exclusion of true positive outliers. Therefore, despite being really similar, we cannot merge the cases where $\left\{d=9,thr_{1}=900,thr_{2}=60\right\}$ and $\left\{d=9,thr_{1}=900,thr_{2}=80\right\}$ by simply choosing the more stringent option. Therefore, we considered combinations that provided not only the best optimization metrics, but also combinations found within 5% of this value (i.e., a list of better choices, instead of simply best choices). Under these conditions, the $\left\{d=9,thr_{1}=900,thr_{2}=80\right\}$ combination, that is the second best option, is found as the better option in 81 out of 100 iterations (table 3). We therefore present in table 4 the power, false positive rate and proportion of false positives calculated for the remaining simulations (see Materials and Methods) and averaged for the 81 cases where $\left\{d=9,thr_{1}=900,thr_{2}=80\right\}$ was the best set of criteria. Similarly, when using power as the optimizing metric, the better option corresponds to $\left\{d=16,thr_{1}=450,thr_{2}=40\right\}$.

**Table 4.** Ability to Detect the Focal Loci Using the “Optimal” Combination of Criteria: $\left\{d=9,thr_{1}=900,thr_{2}=80\right\}$, as Determined by the Cross-Validation Analysis for the Various Scenarios.

(*A*) Scenario	Prop. False Pos. in Outliers	Power	False Pos. Rate
Default	0.017	0.613	$4.1\times 10^{-5}$
Default rec.	0.018	0.436	$3.1\times 10^{-5}$
High ep.	0.062	0.696	$1.8\times 10^{-4}$
High ep. rec.	0.032	0.633	$7.4\times 10^{-5}$
Low mig.	0.042	0.734	$1.4\times 10^{-4}$
Low mig. rec.	0.036	0.659	$9.9\times 10^{-5}$
SL-sel and ep.	0.145	0.485	$4.2\times 10^{-4}$
SL-sel and ep. rec.	0.021	0.202	$2.9\times 10^{-5}$

(*B*) Scenario	Prop. False Pos. in Outliers	Power	False Pos. Rate

Neutral	0.013	0	$1.30\times 10^{-5}$
SL-sel.	0.11	0.350	$1.73\times 10^{-5}$
High SL-sel.	0.178	0.671	$3.9\times 10^{-4}$
Low ep.	$2.50\times 10^{-3}$	0.124	$2.60\times 10^{-6}$
Low ep. rec.	$6.25\times 10^{-3}$	0.068	$7.80\times 10^{-6}$
High mig.	0.01	$6.25\times 10^{-3}$	$1.04\times 10^{-5}$
High mig. rec.	$2.50\times 10^{-3}$	0.0125	$2.60\times 10^{-6}$
High mig high sel.	0.087	0.134	$1.17\times 10^{-4}$
High mig high sel. rec.	0.033	$5.88\times 10^{-2}$	$3.38\times 10^{-5}$

Note.—The correction for multiple testing is a factor 9/100, meaning that the false positive rate should be below $4.5\times 10^{-3}$. (*A*) Scenarios used during the cross-validations and therefore corresponds to the average over the 81 cases where {9, 900, 80} was the best combination or within a 5% distance of it. (*B*) Other scenarios.

As displayed in table 4, using the common set of criteria determined above, we can detect incompatibility loci over a broad range of scenarios with a resolution of 9.5 Mb. As long as epistatic selection is not too weak, we are able to detect the loci with power near 50%, whereas maintaining a false positive rate below 5% (corrected for multiple testing, see Materials and Methods), for both recessive incompatibilities and codominant incompatibilities. The proportion of false positives in outliers fluctuates more but remains below 5% in all but two scenarios: 1) strong codominant epistatic selection and 2) antagonistic single-locus and codominant epistatic selection (table 4*A*). For both scenarios, this excess of false positive in outliers is mainly due to the distortion generated by selection extending beyond what is defined as “true positive” (supplementary figs. S8 and S16, Supplementary Material online). This “too large” distortion is either due to the strength of epistatic selection itself or the antagonist interaction between single-locus and epistatic selection maintaining one of the two incompatible allele at high frequency (0.77 after generation 1,000). Our approach fails to detect the incompatibility loci when migration is high.

Single-locus selection is similarly detectable (table 4*B*). About 65% of loci under strong single-locus selection are detected, a rate similar to the strong epistatic selection scenario. Weaker single-locus selection is detected only about 35% of the time.

When both epistatic selection and single-locus selection (antagonistically) affect the two loci, power decreases from 61% to 49% in the codominant case and from 44% to 20% in the recessive one (table 4*A*), compared with epistatic selection acting in isolation. Using a common combination of $\left\{d,thr_{1},thr_{2}\right\}$ and the power/(2*d+1) optimization metric does not reduce our ability to detect this scenario in the codominant case (table 2) but it does for the recessive case.

When using power as the optimizing metric, the best combination of criteria is $\left\{d=16,thr_{1}=400,thr_{2}=40\right\}$ and the power achieved is far greater than when using power/(2*d+1) as the optimizing metric, reaching above 95% in some cases (supplementary table S2, Supplementary Material online). This increase comes at the cost of lower resolution (16.5 vs. 9.5 Mb). Only the scenarios with strong migration and default epistatic selection perform poorly. In the absence of epistatic selection, the focal loci are detected in 3.25% of the cases, which is less than expected assuming the position of outliers is independently and uniformly distributed along the chromosome (6.8%). A total of 172 outliers are found in 130 of the 400 cases, but in 79 of the 100 replicates. The distribution of outliers detected across the 100 replicates for each of the time samples can be found in supplementary figure S20, Supplementary Material online.

To gauge the applicability of our method to hybrid zones with different ages, we examined performance at the four time points at which we sampled the hybrid population. For most scenarios, power is similar across time points (supplementary figs. S21−S24, Supplementary Material online). This result indicates that our method is robust to variation in hybrid zone age and it can be applied without assumptions about this parameter.

### Stronger Epistatic Selection Is Detected Further Away

**Fig. 4.**
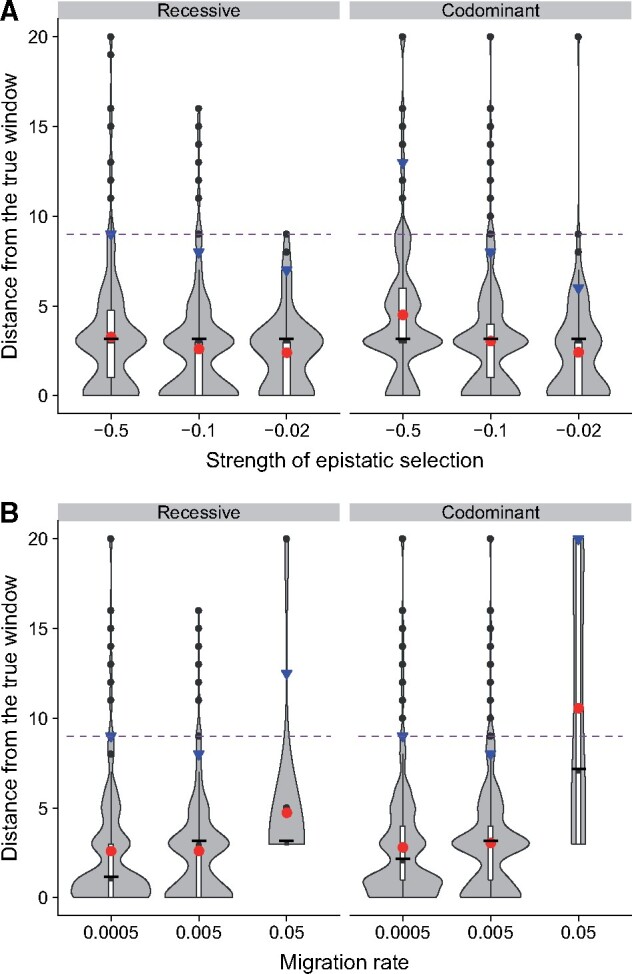
Distribution of distances between the detected outliers and the incompatible loci. Outliers were determined using the following filters $\left\{d=9,thr_{1}=900,thr_{2}=80\right\}$. For both panels, points found more than 20 windows away (10 Mb) were removed, as they are unlikely to reflect selection. Mean distance is given by the red circle, median by the thick black line and the 95^th^ percentile by the blue triangle. The purple dashed line corresponds to *d *=* *9 and separate “true positive” from “false positive.” (*A*) Distance between the detected outliers and the incompatibility loci for different strengths of the incompatibility with the default migration rate, *m *=* *0.005. (*B*) Distance between the detected outliers and the incompatibility loci for different migration rate with the default epistasis coefficient, ϵ=-0.1.

When epistatic selection is strong, the focal outlier is usually detected further away from the positions of incompatibility loci (fig. 4*A*; this is also true for single-locus selection in supplementary fig. S25, Supplementary Material online). In the absence of selection, the SFS is bimodal, with one mode corresponding to rare alleles and the second mode near 0.5 due to fixed differences between the two parental populations (fig. 1*D*). Selection distorts the local SFS (fig. 2*A*; supplementary figs. S3−S17, Supplementary Material online); alleles with frequencies at or near 0.5 are more strongly affected because many of them are in tight linkage disequilibrium with incompatibility alleles. About half of the mutations at frequency 0.5 originated from population 1 and are in strong linkage disequilibrium with allele *A* (or *b*), whereas the other half originated from population 2 and are in strong association with allele *a* (or *B*). Due to epistatic selection, allele *A* decreases in frequency, and so do the neutral alleles in linkage disequilibrium with *A*. Conversely, allele *a* and linked neutral alleles increase in frequency. This dynamic splits in two parts the mode of the SFS near 0.5. When selection is strong, neutral alleles in linkage disequilibrium with A are pushed to a lower frequency, making it harder to distinguish the SFS under strong epistatic selection from the baseline SFS. Therefore, the signal is recovered further away. Put another way, incompatibility alleles decrease in frequency at a faster rate compared with recombination when selection is strong. As a result, linkage disequilibrium between incompatibility alleles and neutral alleles extends further and so does the distortion in the SFS.

We recover a similar pattern for migration (fig. 4*B*). With stronger migration, the detected outliers are on average further away from the positions of incompatibility loci because a high proportion of incompatibility alleles have been recently introduced into the hybrid population and linked neutral alleles have had less time to recombine away. However, migration and epistatic selection seem to interact nonlinearly. Indeed, for a migration rate of *m *=* *0.05, incompatibility loci under strong epistatic selection are detected closer to their true positions (supplementary fig. S26, Supplementary Material online) than are incompatibility loci under weak epistatic selection. Finally, in the presence of antagonistic single-locus selection, the detected outliers are on average further away from the positions of incompatibility loci (supplementary fig. S27, Supplementary Material online).

## Discussion

Although hybrid incompatibilities are important contributors to reproductive isolation and speciation, they remain challenging to identify. We have shown that epistatic selection against incompatibility loci produces a localized distortion in the SFS in hybrid populations. Using this distortion, we can identify chromosomal regions that contain incompatibility loci under a variety of scenarios.

Our approach features several advantages. First, the method is applicable to any pair of nascent species that form a natural hybrid zone; breeding organisms in the laboratory is not required. Second, our strategy can detect incompatibility loci that affect any aspect of hybrid viability or hybrid fertility without specifying the phenotype of interest in advance. In addition, the method performs relatively well across a wide range of epistatic selective strength. Third, using the genome-wide SFS to predict the local SFS accounts for effects of demographic history, obviating the need to reconstruct the history of hybridization (which can be complex). Fourth, many of the known examples of incompatible genes involve strongly diverged species (Powell et al. 2020), presenting a risk that they arose after the speciation process was complete. The method we described relies on individuals from natural hybrid zones. Genetic incompatibilities detected are therefore likely to be involved in the speciation process. Finally, our approach incorporates the full SFS and is specifically designed for the analysis of genome sequence data. Below, we discuss the application and limits of the method.

One of the biggest challenges in the detection of hybrid incompatibilities is disentangling epistatic selection from single-locus selection. Although disrupted interactions between alleles at two loci reducing hybrid fitness and a beneficial allele at a single-locus increasing fitness are biologically distinct phenomena, they leave similar signatures in the SFS. Selection against an incompatibility (derived) allele increases the frequency of the nonincompatibility (ancestral) allele in a manner that resembles a selective sweep involving a single-locus. Epistatic selection is less effective than single-locus selection because an incompatibility allele is only disfavored when it is combined with the incompatibility allele at the second locus (this difference is strongest when epistatic interactions are fully recessive). As a result, we might expect epistatic selection to be detected only when it is strong. In contrast, epistatic selection leading to a fitness reduction of 10% in double heterozygotes is easily detectable using our method. Cases with even weaker epistatic selection can be detected with limited power, depending in part on the balance between resolution and power that is chosen (table 4; supplementary table S2, Supplementary Material online). Nevertheless, when applied in isolation, our method is not able to exclude the possibility that single-locus selection explains detected outlier loci.

We suggest several potential solutions to the challenge of distinguishing epistatic selection from single-locus selection. First, clines in allele frequency or genotype frequency at SNPs near detected outliers can be examined in a second stage of analysis. An explicit epistatic model can be fit using genomic clines (Gompert and Buerkle 2009, 2011). If samples from additional localities in the hybrid zone can be collected, geographic clines can be analyzed for evidence of adaptation of the form that would be predicted under single-locus selection (Endler 1977). Second, longitudinal data from hybrid zones have the potential to detect differences in the trajectory of alleles under epistatic selection and single-locus selection. Allele trajectories have started to be exploited to detect selection (Foll et al. 2015) and to understand the evolution of quantitative traits (Franssen et al. 2017). Finally, we emphasize that we would expect many of the loci identified by our approach to be incompatibilities, at least when considering hybrid zones between lineages with reproductive barriers. The number of hybrid incompatibilities is expected to grow faster than linearly with divergence time (Orr 1995) and this result has been recovered in multiple taxa: Including *Solanum* (Moyle and Nakazato 2010; Guerrero et al. 2017), *Drosophila* (Matute et al. 2010), or *Mus musculus* (Wang et al. 2015).

Our approach offers the potential to identify both loci involved in a hybrid incompatibility. However, because each genomic region is evaluated independently, the current method does not reveal whether detected loci interact epistatically. Schumer and Brandvain (2016) demonstrated that pairs of incompatibility loci tend to be found in the extreme tail of the distribution of admixture disequilibrium, providing a possible way forward. Unfortunately, our preliminary investigation of admixture disequilibrium revealed that this signature is not maintained in older hybrid zones (supplementary figs. S3−S17 and S28, Supplementary Material online). We suspect this disparity reflects our decision to model (BDM) incompatibilities with asymmetric fitness effects (whereas Schumer and Brandvain [2016] focused on incompatibilities with symmetric fitness effects) and to consider scenarios with weaker selection, in addition to the limited time frame during which epistatic selection distorts admixture disequilibrium. In another attempt to detect interactions among selected loci, Beissinger et al. (2016) computed the $D_{\mathrm{IS}}^{\prime 2}$ statistic (Ohta 1982a, 1982b), a measure that partitions linkage disequilibrium between and within populations. Although a certain number of candidate genes were identified, the authors pointed out that single-locus selection could not be excluded as an alternative explanation since selective sweeps can generate similar patterns.

The need to include enough SNPs to formulate a reasonable SFS and the extra steps taken to minimize the detection of false positives jointly reduce the genomic resolution of our method. An incompatibility locus can be associated with a region of about 9 − 15 Mb, depending on whether we optimize for power or resolution. Nevertheless, most outliers are found much closer to the incompatibility locus itself (fig. 4). For example, in the default case we examined, outliers were located within 1.4 Mb of the incompatibility locus on average. The same genome sequence data required for our method can be used to further refine the location of incompatibility loci. We expect incompatibility alleles to be enriched for fixed differences outside the hybrid zone; scanning parental populations for fixed differences or large frequency differences could be a reasonable filter. Genes with relatively high levels of nonsynonymous divergence between parental populations could be prioritized. Searching outlier regions for alleles that show narrow frequency clines could also localize incompatibility loci. Finally, in species with annotated genomes or their relatives, knowledge of gene function could be used to identify candidate genes. For example, genes that reduce fertility when mutated could be good targets for further examination. Alternatively, our method could be used to suggest new classes of genes involved in reproductive isolation by searching for pathways that are enriched across outliers. Confirmation that a gene participates in an incompatibility will ultimately require experimental validation (see, e.g., Bozdag et al. 2021), which is becoming possible for an increasing variety of species through advances in genome editing.

Several biologically realistic scenarios missing from our simulation model deserve consideration. In addition to epistatic selection against hybrid incompatibilities, reduced introgression can result from single-locus selection against deleterious mutations that accumulate in source populations with small effective sizes (Harris and Nielsen 2016). Although our simulations assumed that incompatibility alleles were fixed in source populations, incompatibilities are often polymorphic in nature (Cutter 2012). More complex models of migration including structured source populations, asymmetrical migration, and migration out of hybrid populations are likely features of real hybrid zones. Finally, both hybrid populations and source populations will routinely depart from mutation-drift equilibrium due to demographic factors. Examining the extent to which each of these scenarios produces local distortions in the SFS of hybrid populations is a logical next step.

Our measurement of method performance also assumed that sequences could be determined with complete accuracy. Genotyping error could reduce power and increase the false-positive rate, especially for low-coverage sequencing, which can lead to biased reconstructions of the SFS (Han et al. 2014). We recommend incorporating uncertainty in variant calling when building the SFS from sequencing reads (Nielsen et al. 2012) before applying our approach.

## Supplementary Material

Supplementary data are available at *Molecular Biology and Evolution* online.

## Supplementary Material

msab168_Supplementary_Data
