# Supplementary material for: Finding Hybrid Incompatibilities Using Genome Sequences from Hybrid Populations
Source: Mol Biol Evol. 2021 Jun 7;38(10):4616–27. doi: 10.1093/molbev/msab168 (PMC8476132; doi:10.1093/molbev/msab168)
Supplement: msab168_Supplementary_Data [file msab168_supplementary_data.zip › SI_revised.pdf]

Supplementary Information: Finding hybrid incompatibilities using genome sequences from hybrid populations

Alexandre Blanckaert *[1] and Bret A. Payseur[1]

[1]Laboratory of Genetics, University of Wisconsin-Madison, WI 53706 Madison, United States

May 27, 2021

*blanckaert.a@gmail.com

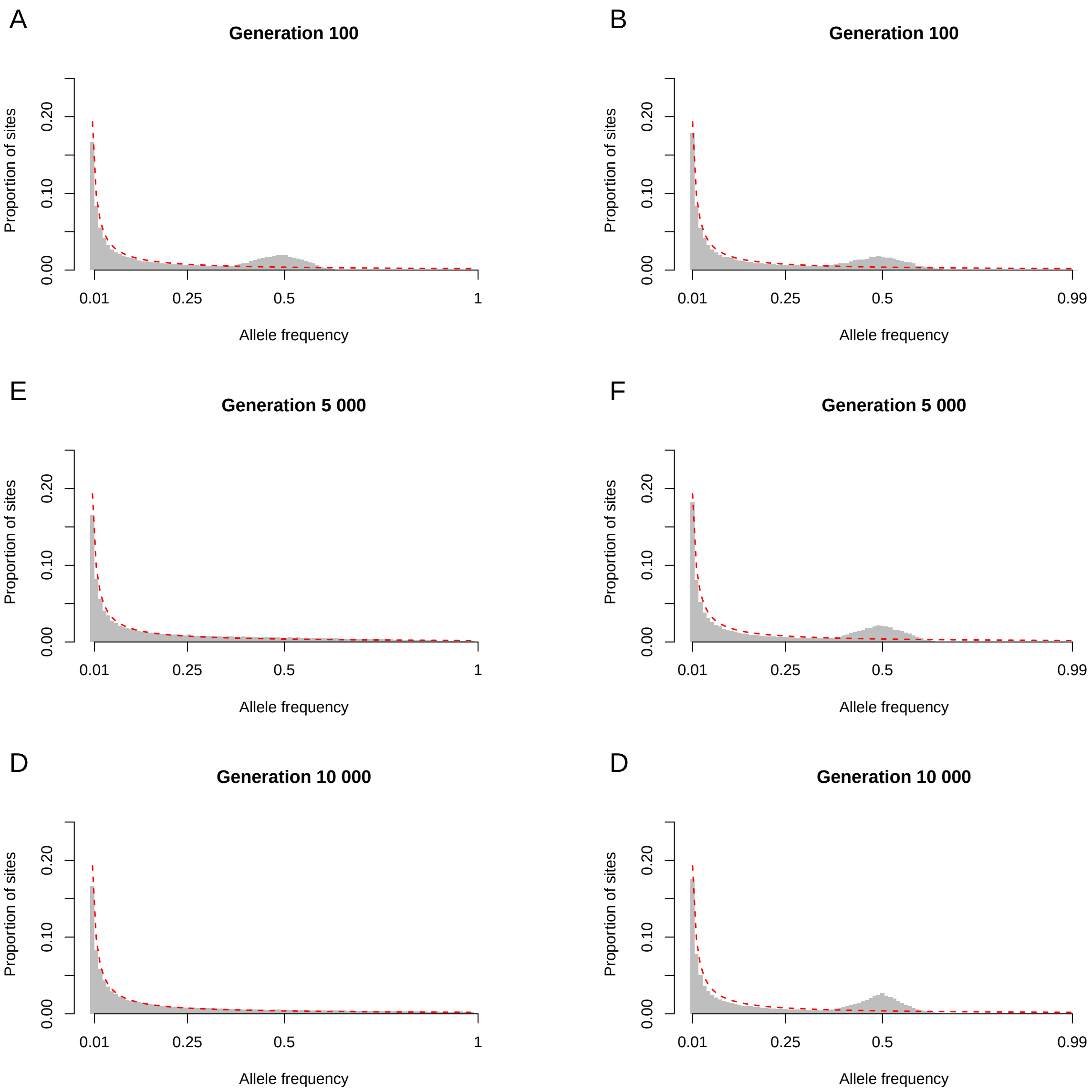

Figure S1: Genome-wide site frequency spectrum for a neutral hybrid population for 3 different time sample (panels A and B *gen.* = 100, panels C and D *gen.* = 5000 and panels E and F *gen.* = 10000. The left panels (A,C,E) corresponds to an isolated hybrid population and the right panels to a hybrid population receiving gene flow from its parental sources ($m = 0.005$). The red dashed line corresponds to the SFS of an isolated population with a similar $N\mu$.) The SFS for generation 1 000 are given in main manuscript, Figure 1

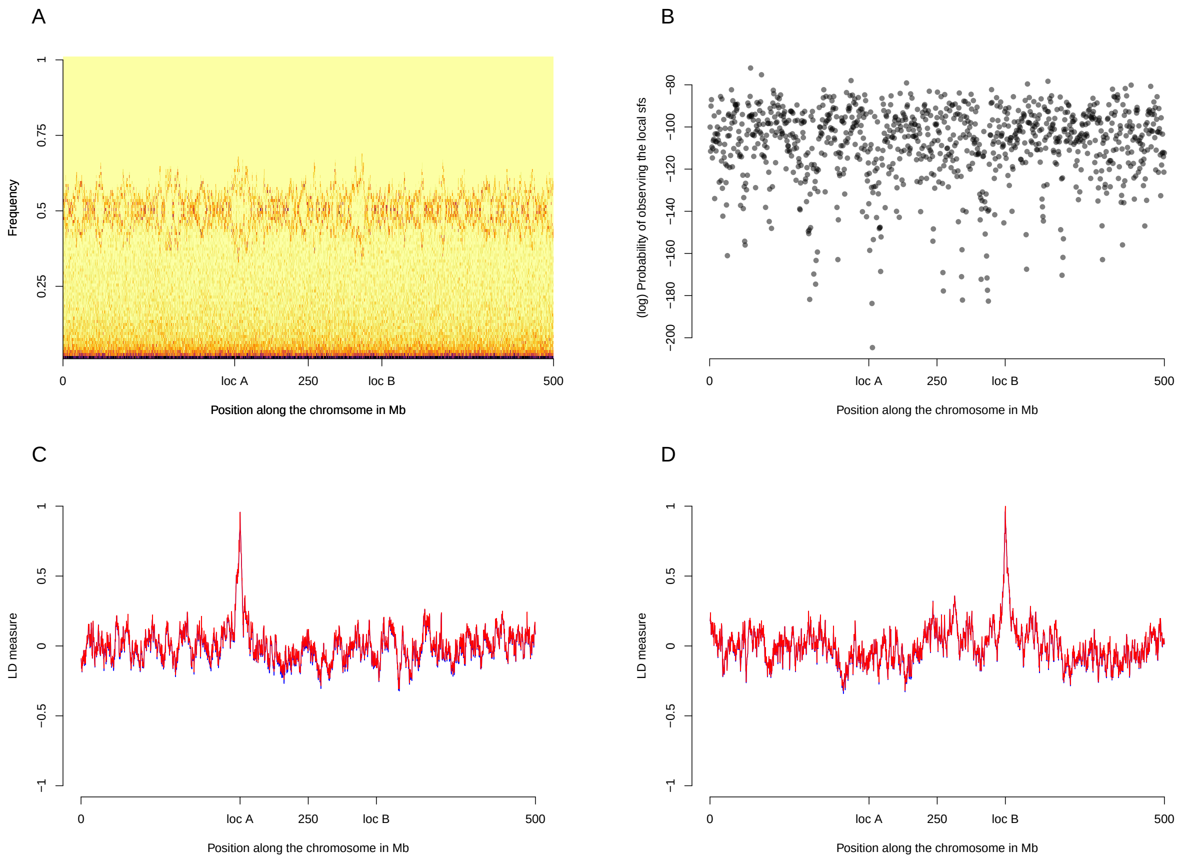

Figure S2: Illustration for the "neutral" scenario. A/ Local site frequency spectrum calculated over regions of 100kb. The X axis corresponds to the position along the chromosome, the Y axis to the frequency and the density is given by color, with yellow denoting a lack of SNPs with the corresponding frequency and black an abundance of them. B/ Probability of observing the local site frequency spectrum based on the global site frequency spectrum. C (resp. D)/ Linkage disequilibrium between locus A (resp. locus B) and the whole chromosome using uniquely fixed differences between the two parental population, calculated between alleles fixed in the same population (in red, the correlation coefficient, and in blue, the partial correlation given the genome-wide ancestry proportion). The LD between alleles A and B has therefore the opposite sign and is given by $r_{AB} = 0.156$ for the correlation coefficient and $r_{|a,AB} = 0.157$ for the partial correlation given the genome-wide ancestry proportion. Parameters used: $s = 0, \epsilon = 0, m = 0.005, gen. = 1,000$

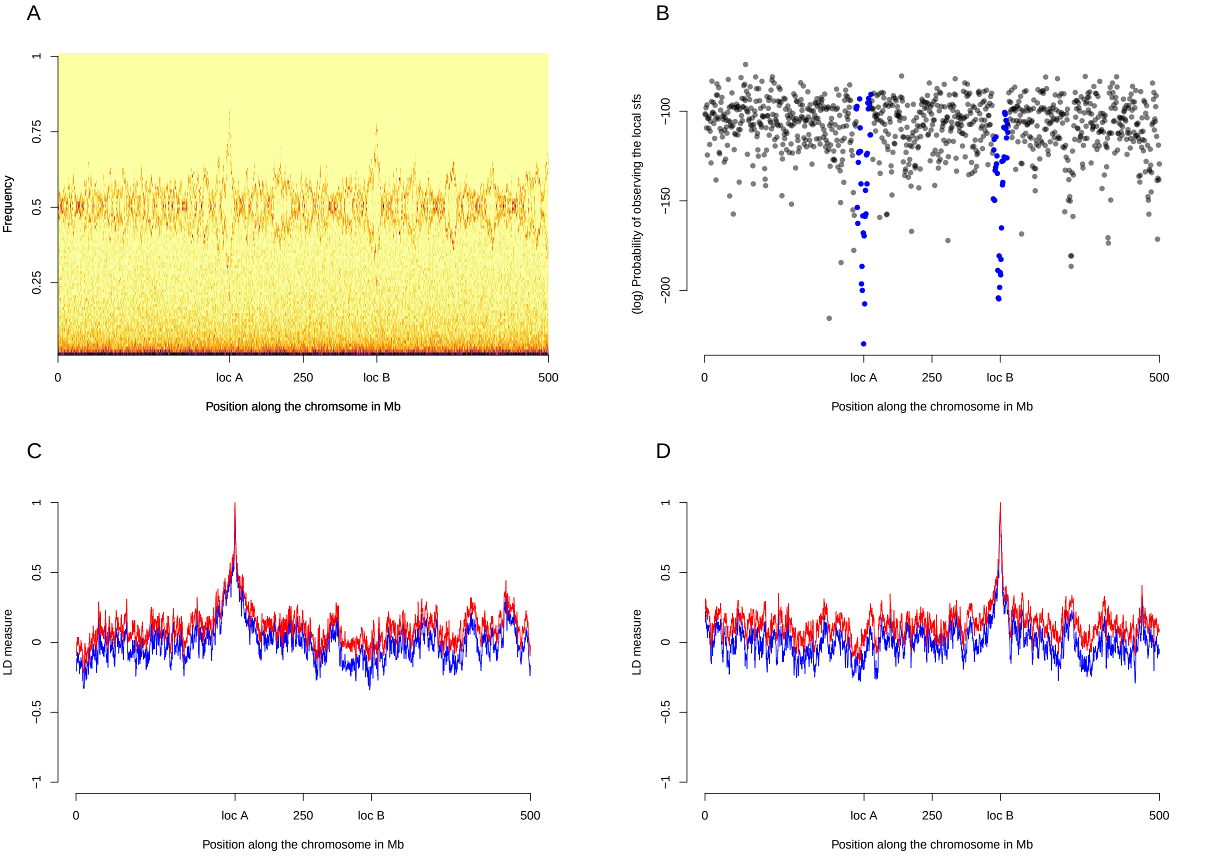

Figure S3: Illustration for the "single locus selection" scenario.A/ Local site frequency spectrum calculated over regions of 100kb. The X axis corresponds to the position along the chromosome, the Y axis to the frequency and the density is given by color, with yellow denoting a lack of SNPs with the corresponding frequency and black an abundance of them. B/ Probability of observing the local site frequency spectrum based on the global site frequency spectrum. Windows considered as "true positive" (based on Table S1, 16 on each side of A and B) are colored in blue. C (resp. D)/ Linkage disequilibrium between locus A (resp. locus B) and the whole chromosome using uniquely fixed differences between the two parental population, calculated between alleles fixed in the same population (in red, the correlation coefficient, and in blue, the partial correlation given the genome-wide ancestry proportion). The LD between alleles A and B has therefore the opposite sign and is given by $r_{AB} = 0.0147$ for the correlation coefficient and $r_{|a,AB} = 0.192$ for the partial correlation given the genome-wide ancestry proportion. Parameters used: $s = -0.02, \epsilon = 0, m = 0.005, gen. = 1,000$

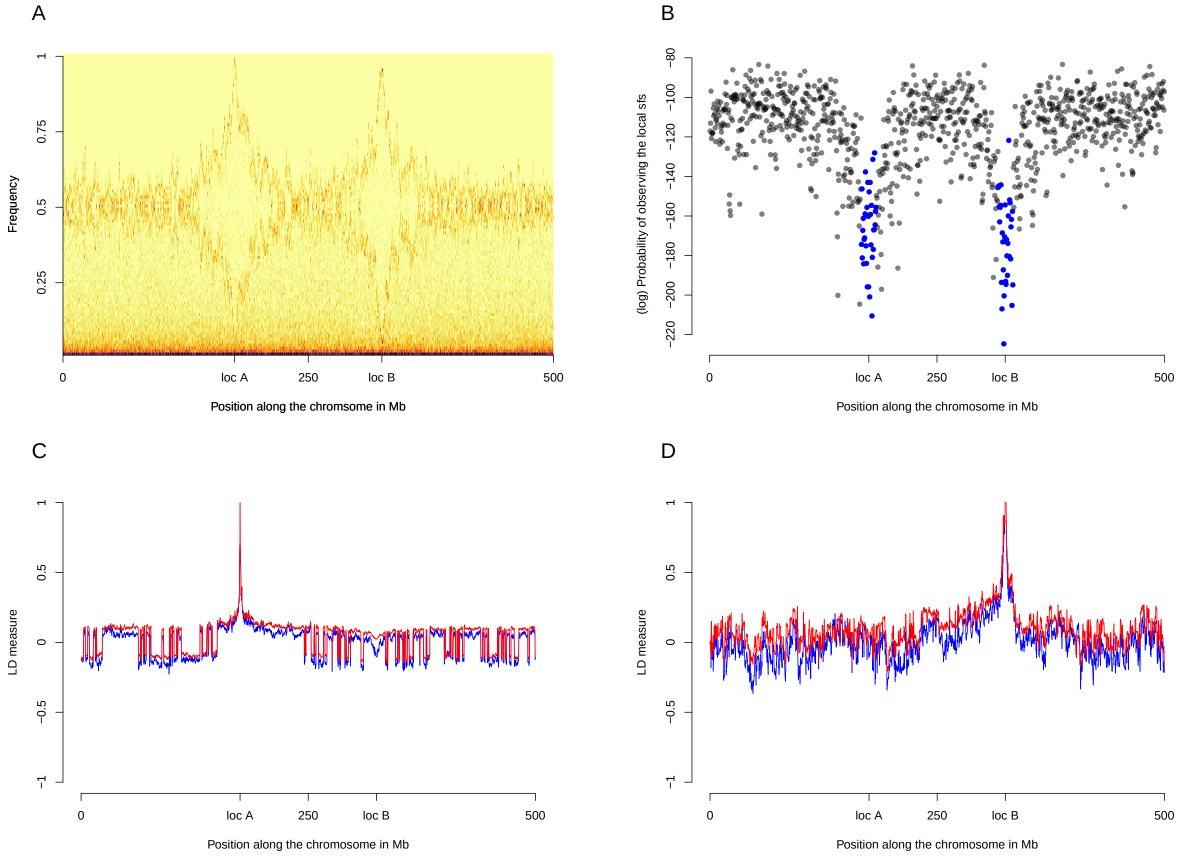

Figure S4: Illustration for the "high single locus selection" scenario. A/ Local site frequency spectrum calculated over regions of 100kb. The X axis corresponds to the position along the chromosome, the Y axis to the frequency and the density is given by color, with yellow denoting a lack of SNPs with the corresponding frequency and black an abundance of them. B/ Probability of observing the local site frequency spectrum based on the global site frequency spectrum. Windows considered as "true positive" (based on Table S1, 16 on each side of A and B) are colored in blue. C (resp. D)/Linkage disequilibrium between locus A (resp. locus B) and the whole chromosome using uniquely fixed differences between the two parental population, calculated between alleles fixed in the same population (in red, the correlation coefficient, and in blue, the partial correlation given the genome-wide ancestry proportion). The LD between alleles A and B has therefore the opposite sign and is given by $r_{AB} = -0.0231$ for the correlation coefficient and $r_{|a,AB} = 0.105$ for the partial correlation given the genome-wide ancestry proportion. Parameters used: $s = -0.1, \epsilon = 0, m = 0.005, gen. = 1,000$

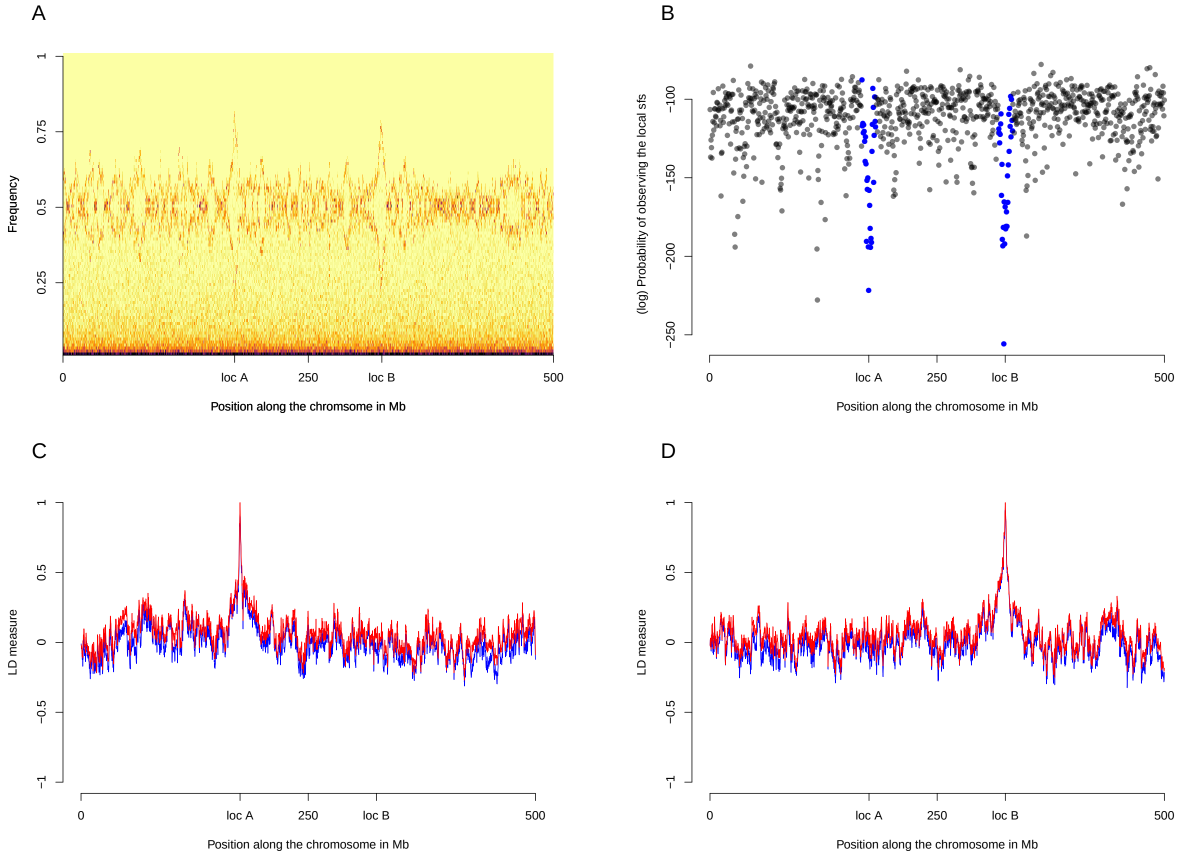

Figure S5: Illustration for the "default recessive" scenario. A/ Local site frequency spectrum calculated over regions of 100kb. The X axis corresponds to the position along the chromosome, the Y axis to the frequency and the density is given by color, with yellow denoting a lack of SNPs with the corresponding frequency and black an abundance of them. B/ Probability of observing the local site frequency spectrum based on the global site frequency spectrum. Windows considered as "true positive" (based on Table 2, 15 on each side of A and B) are colored in blue. C (resp. D)/ Linkage disequilibrium between locus A (resp. locus B) and the whole chromosome using uniquely fixed differences between the two parental population, calculated between alleles fixed in the same population (in red, the correlation coefficient, and in blue, the partial correlation given the genome-wide ancestry proportion). The LD between alleles A and B has therefore the opposite sign and is given by $r_{AB} = -0.0546$ for the correlation coefficient and $r_{|a,AB} = 0.0497$ for the partial correlation given the genome-wide ancestry proportion. Parameters used: $s = 0, \epsilon = -0.1, m = 0.005, gen. = 1,000$

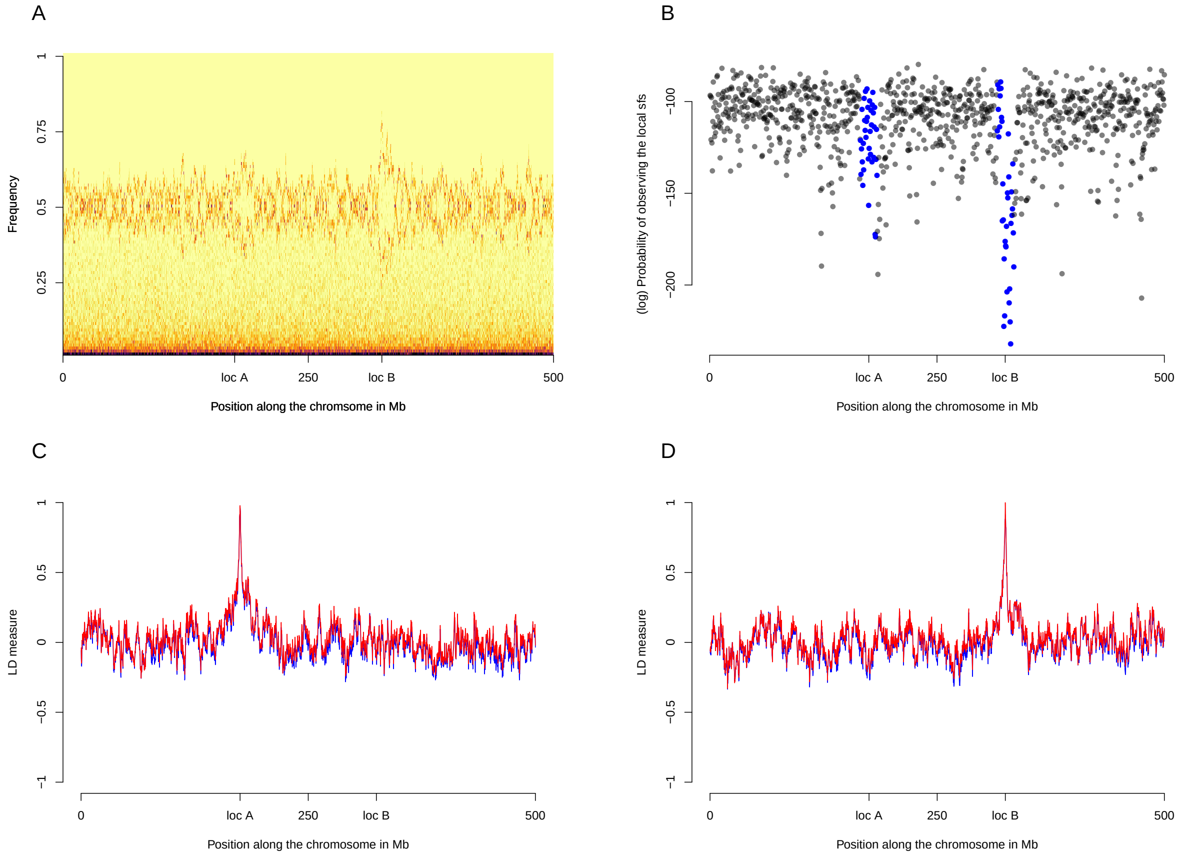

Figure S6: Illustration for the "low epistasis" scenario. A/ Local site frequency spectrum calculated over regions of 100kb. The X axis corresponds to the position along the chromosome, the Y axis to the frequency and the density is given by color, with yellow denoting a lack of SNPs with the corresponding frequency and black an abundance of them. B/ Probability of observing the local site frequency spectrum based on the global site frequency spectrum. Windows considered as "true positive" (based on Table 2, 18 on each side of A and B) are colored in blue. C (resp. D)/ Linkage disequilibrium between locus A (resp. locus B) and the whole chromosome using uniquely fixed differences between the two parental population, calculated between alleles fixed in the same population (in red, the correlation coefficient, and in blue, the partial correlation given the genome-wide ancestry proportion). The LD between alleles A and B has therefore the opposite sign and is given by $r_{AB} = 0.0524$ for the correlation coefficient and $r_{|a,AB} = 0.0787$ for the partial correlation given the genome-wide ancestry proportion. Parameters used: $s = 0, \epsilon = -0.1, m = 0.0005, gen. = 1,000$

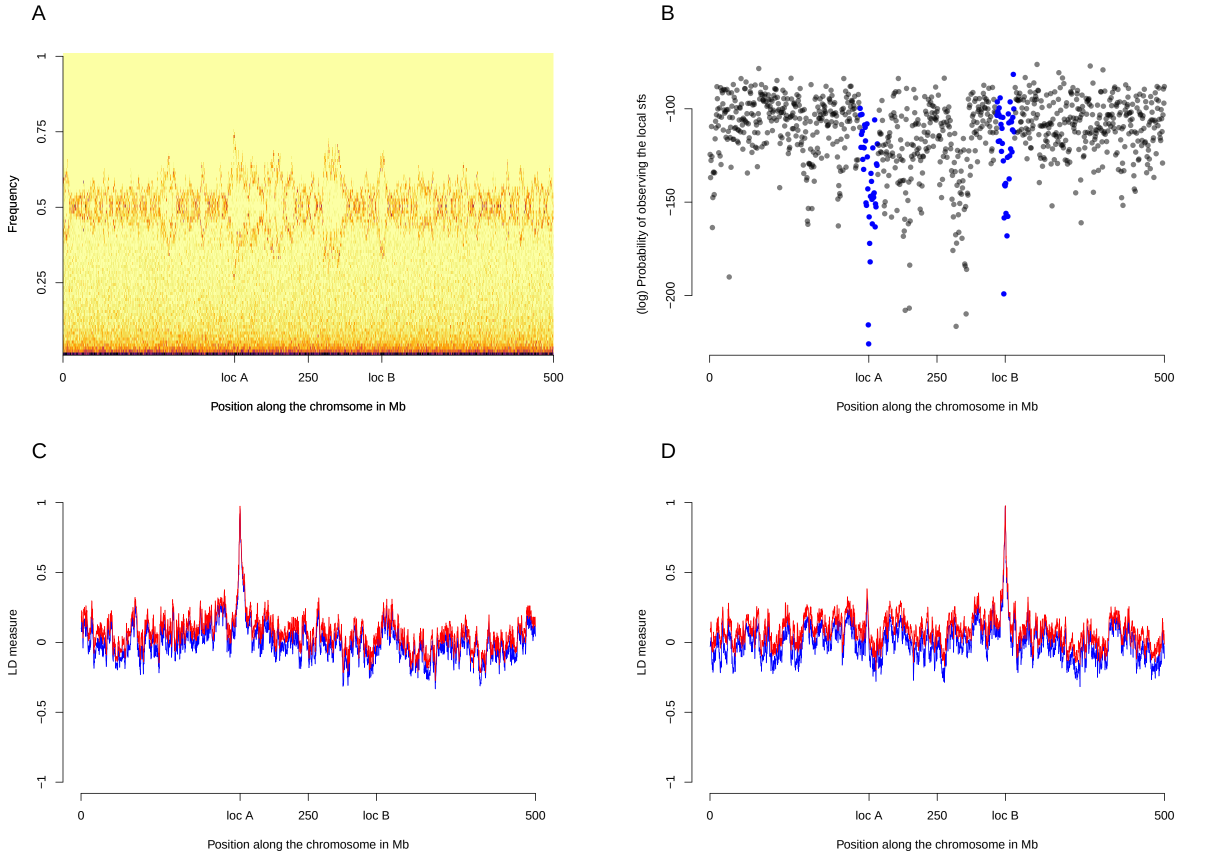

Figure S7: Illustration for the "low epistasis" scenario. A/ Local site frequency spectrum calculated over regions of 100kb. The X axis corresponds to the position along the chromosome, the Y axis to the frequency and the density is given by color, with yellow denoting a lack of SNPs with the corresponding frequency and black an abundance of them. B/ Probability of observing the local site frequency spectrum based on the global site frequency spectrum. Windows considered as "true positive" (based on Table 2, 19 on each side of A and B) are colored in blue. C (resp. D)/ Linkage disequilibrium between locus A (resp. locus B) and the whole chromosome using uniquely fixed differences between the two parental population, calculated between alleles fixed in the same population (in red, the correlation coefficient, and in blue, the partial correlation given the genome-wide ancestry proportion). The LD between alleles A and B has therefore the opposite sign and is given by $r_{AB} = -0.0404$ for the correlation coefficient and $r_{|a,AB} = 0.0426$ for the partial correlation given the genome-wide ancestry proportion. Parameters used: $s = 0, \epsilon = -0.1, m = 0.0005, gen. = 1,000$

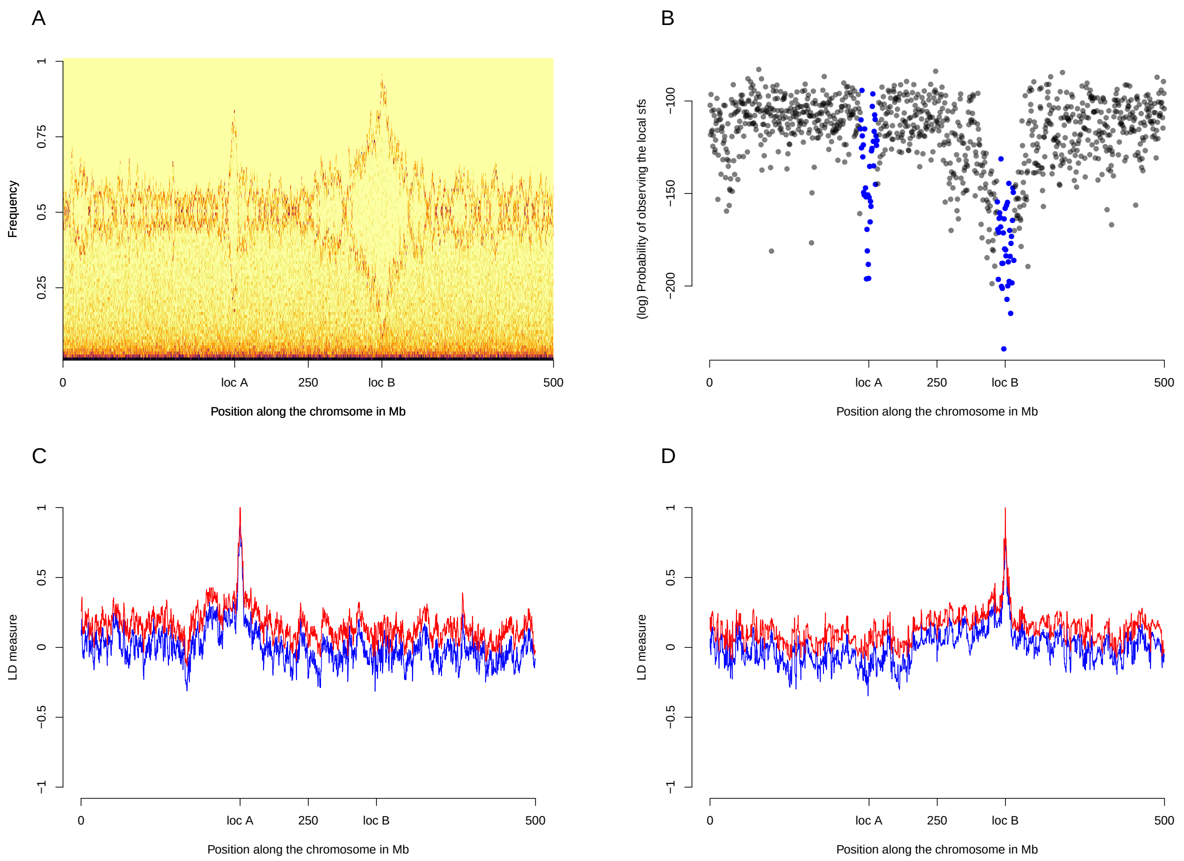

Figure S8: Illustration for the "high epistatic codominant selection" scenario. A/ Local site frequency spectrum calculated over regions of 100kb. The X axis corresponds to the position along the chromosome, the Y axis to the frequency and the density is given by color, with yellow denoting a lack of SNPs with the corresponding frequency and black an abundance of them. B/ Probability of observing the local site frequency spectrum based on the global site frequency spectrum. Windows considered as "true positive" (based on Table 2, 18 on each side of A and B) are colored in blue. C (resp. D)/ Linkage disequilibrium between locus A (resp. locus B) and the whole chromosome using uniquely fixed differences between the two parental population, calculated between alleles fixed in the same population (in red, the correlation coefficient, and in blue, the partial correlation given the genome-wide ancestry proportion). The LD between alleles A and B has therefore the opposite sign and is given by $r_{AB} = -0.104$ for the correlation coefficient and $r_{|a,AB} = 0.148$ for the partial correlation given the genome-wide ancestry proportion. Parameters used: $s = 0, \epsilon = -0.5, m = 0.005, gen. = 1,000$

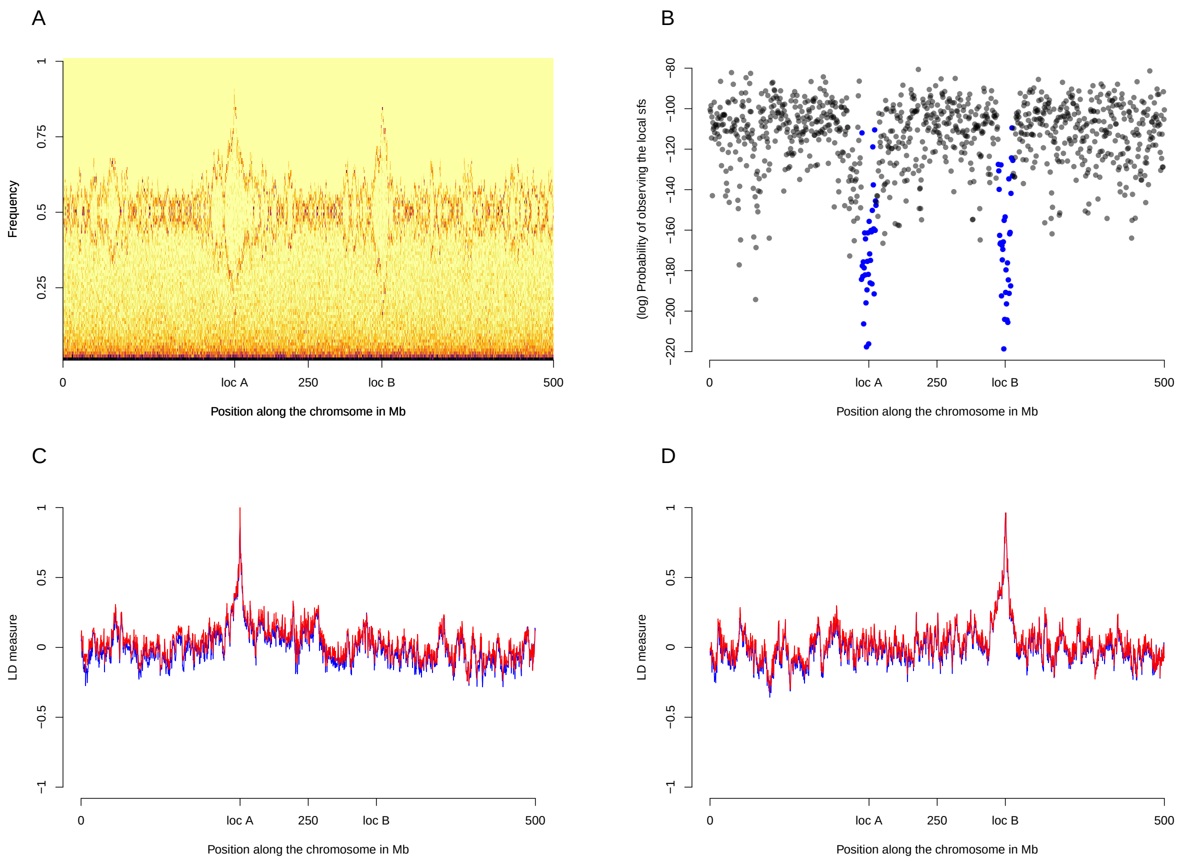

Figure S9: Illustration for the "high epistatic recessive selection" scenario. A/ Local site frequency spectrum calculated over regions of 100kb. The X axis corresponds to the position along the chromosome, the Y axis to the frequency and the density is given by color, with yellow denoting a lack of SNPs with the corresponding frequency and black an abundance of them. B/ Probability of observing the local site frequency spectrum based on the global site frequency spectrum. Windows considered as "true positive" (based on Table 2, 16 on each side of A and B) are colored in blue. C (resp. D)/ Linkage disequilibrium between locus A (resp. locus B) and the whole chromosome using uniquely fixed differences between the two parental population, calculated between alleles fixed in the same population (in red, the correlation coefficient, and in blue, the partial correlation given the genome-wide ancestry proportion). The LD between alleles A and B has therefore the opposite sign and is given by $r_{AB} = -0.0467$ for the correlation coefficient and $r_{|a,AB} = -0.0226$ for the partial correlation given the genome-wide ancestry proportion. Parameters used: $s = 0, \epsilon = -0.5, m = 0.005, gen. = 1,000$

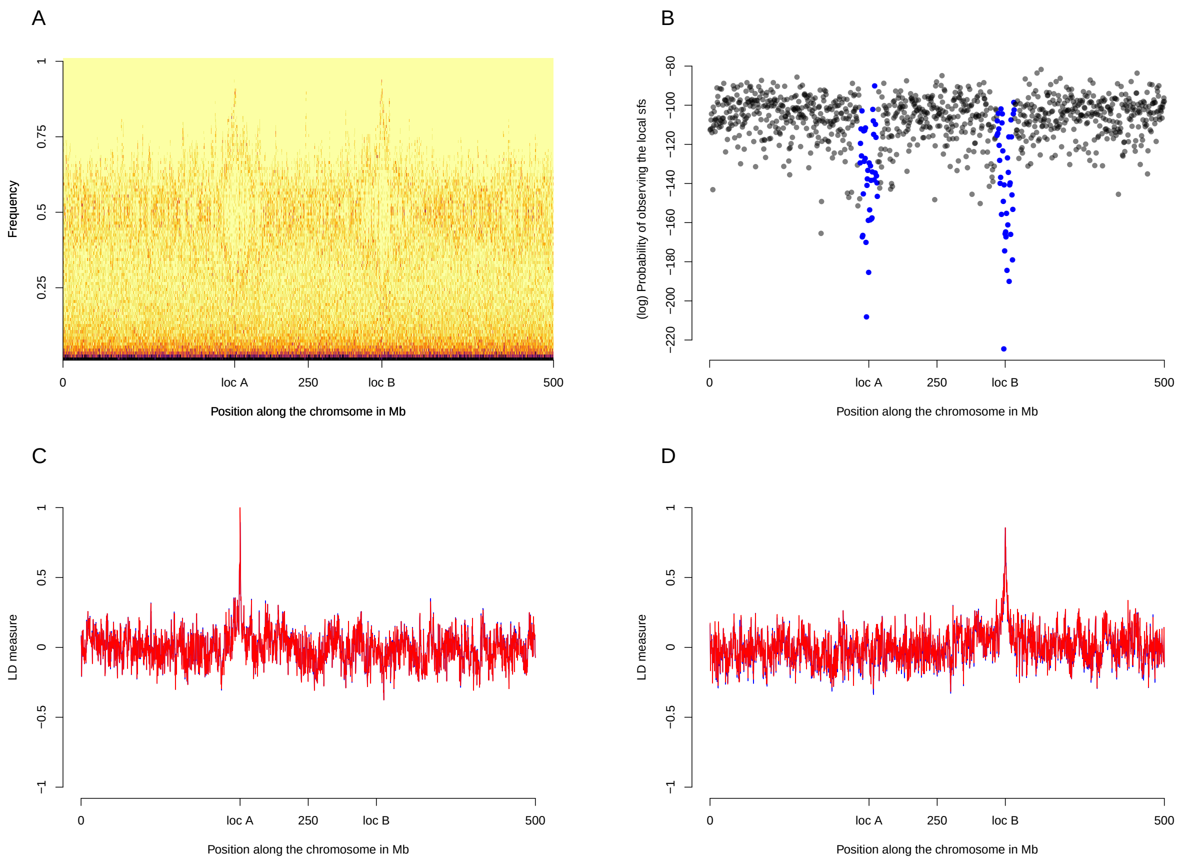

Figure S10: Illustration for the "low migration" scenario. A/ Local site frequency spectrum calculated over regions of 100kb. The X axis corresponds to the position along the chromosome, the Y axis to the frequency and the density is given by color, with yellow denoting a lack of SNPs with the corresponding frequency and black an abundance of them. B/ Probability of observing the local site frequency spectrum based on the global site frequency spectrum. Windows considered as "true positive" (based on Table 2, 19 on each side of A and B) are colored in blue. C (resp. D)/ Linkage disequilibrium between locus A (resp. locus B) and the whole chromosome using uniquely fixed differences between the two parental population, calculated between alleles fixed in the same population (in red, the correlation coefficient, and in blue, the partial correlation given the genome-wide ancestry proportion). The LD between alleles A and B has therefore the opposite sign and is given by $r_{AB} = 0.0677$ for the correlation coefficient and $r_{|a,AB} = 0.0625$ for the partial correlation given the genome-wide ancestry proportion. Parameters used: $s = 0, \epsilon = -0.1, m = 0.0005, gen. = 1,000$

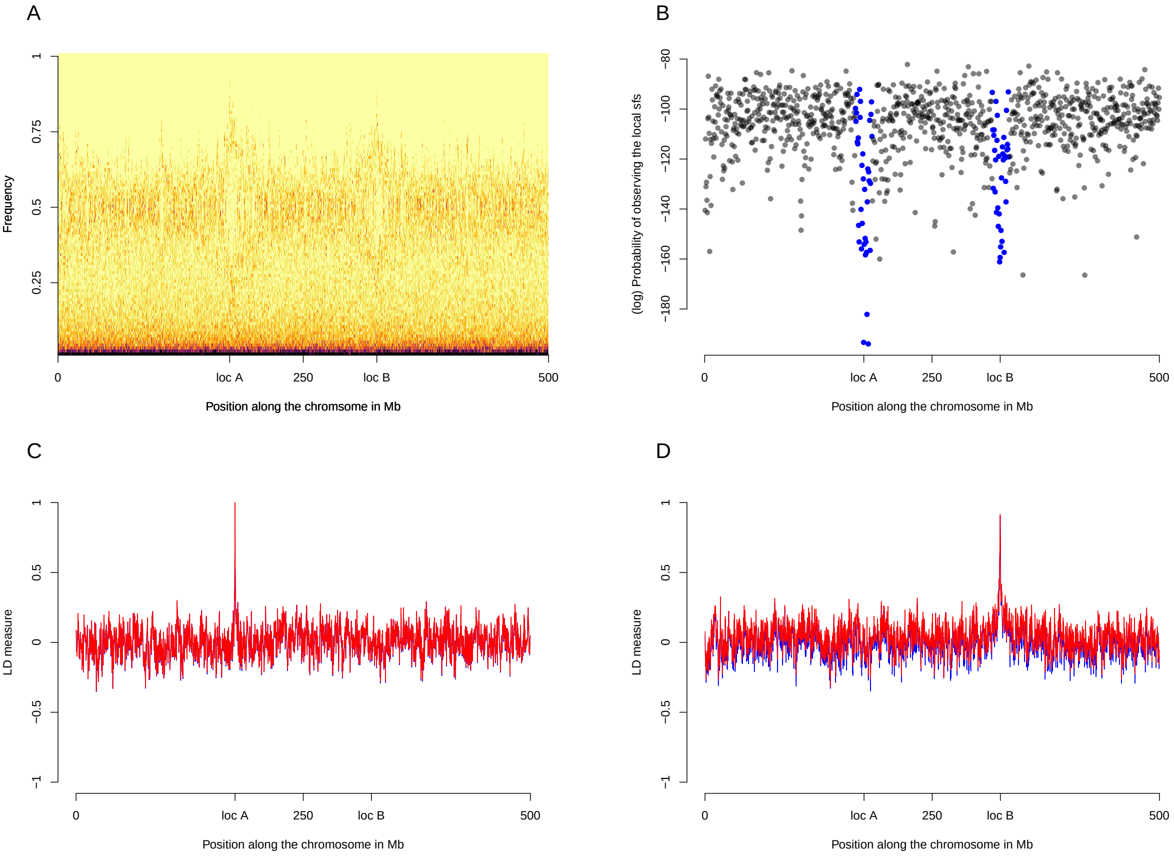

Figure S11: Illustration for the "low migration recessive" scenario. A/ Local site frequency spectrum calculated over regions of 100kb. The X axis corresponds to the position along the chromosome, the Y axis to the frequency and the density is given by color, with yellow denoting a lack of SNPs with the corresponding frequency and black an abundance of them. B/ Probability of observing the local site frequency spectrum based on the global site frequency spectrum. Windows considered as "true positive" (based on Table 2, 18 on each side of A and B) are colored in blue. C (resp. D)/ Linkage disequilibrium between locus A (resp. locus B) and the whole chromosome using uniquely fixed differences between the two parental population, calculated between alleles fixed in the same population (in red, the correlation coefficient, and in blue, the partial correlation given the genome-wide ancestry proportion). The LD between alleles A and B has therefore the opposite sign and is given by $r_{AB} = 0.0284$ for the correlation coefficient and $r_{|a,AB} = 0.0435$ for the partial correlation given the genome-wide ancestry proportion. Parameters used: $s = 0, \epsilon = -0.1, m = 0.0005, gen. = 1,000$

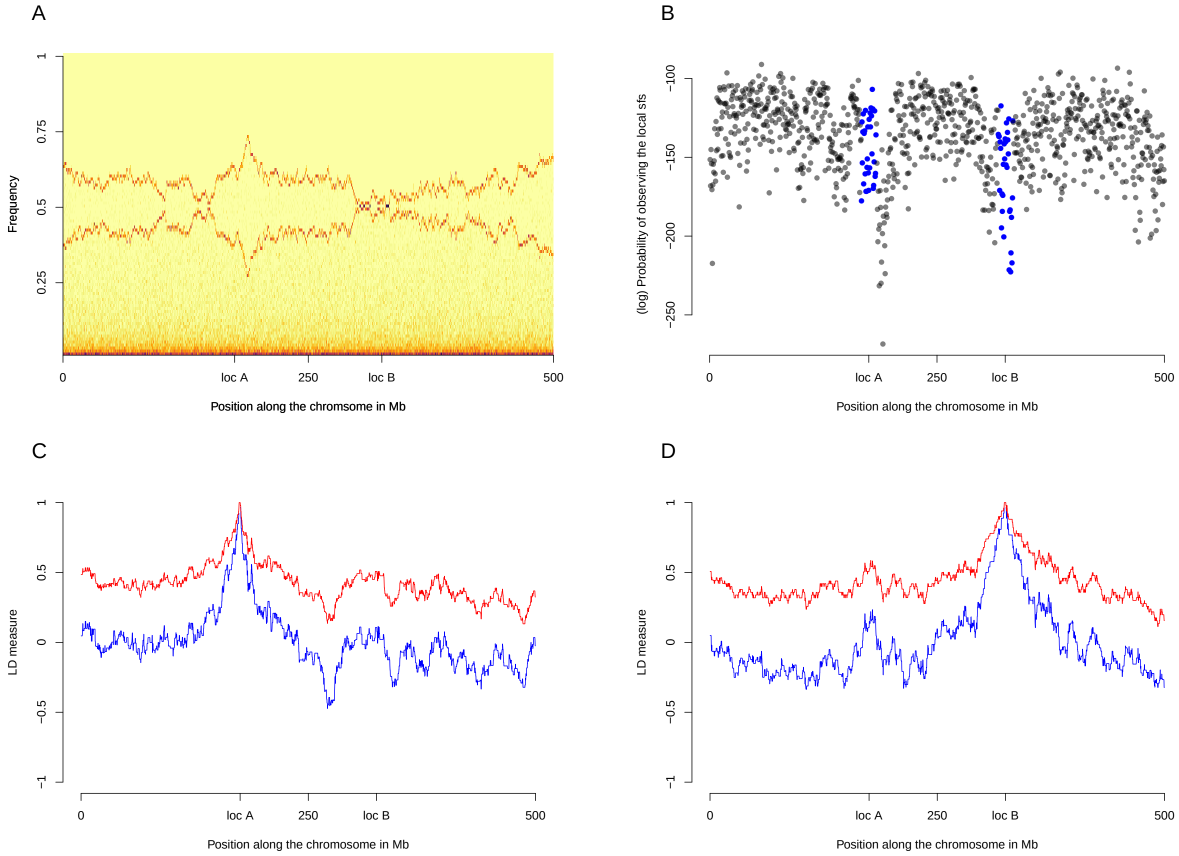

Figure S12: Illustration for the "high migration" scenario. A/ Local site frequency spectrum calculated over regions of 100kb. The X axis corresponds to the position along the chromosome, the Y axis to the frequency and the density is given by color, with yellow denoting a lack of SNPs with the corresponding frequency and black an abundance of them. B/ Probability of observing the local site frequency spectrum based on the global site frequency spectrum. Windows considered as "true positive" (based on Table 2, 16 on each side of A and B) are colored in blue. C (resp. D)/ Linkage disequilibrium between locus A (resp. locus B) and the whole chromosome using uniquely fixed differences between the two parental population, calculated between alleles fixed in the same population (in red, the correlation coefficient, and in blue, the partial correlation given the genome-wide ancestry proportion). The LD between alleles A and B has therefore the opposite sign and is given by $r_{AB} = -0.507$ for the correlation coefficient and $r_{|a,AB} = -0.106$ for the partial correlation given the genome-wide ancestry proportion. Parameters used: $s = 0, \epsilon = -0.1, m = 0.05, gen. = 1,000$

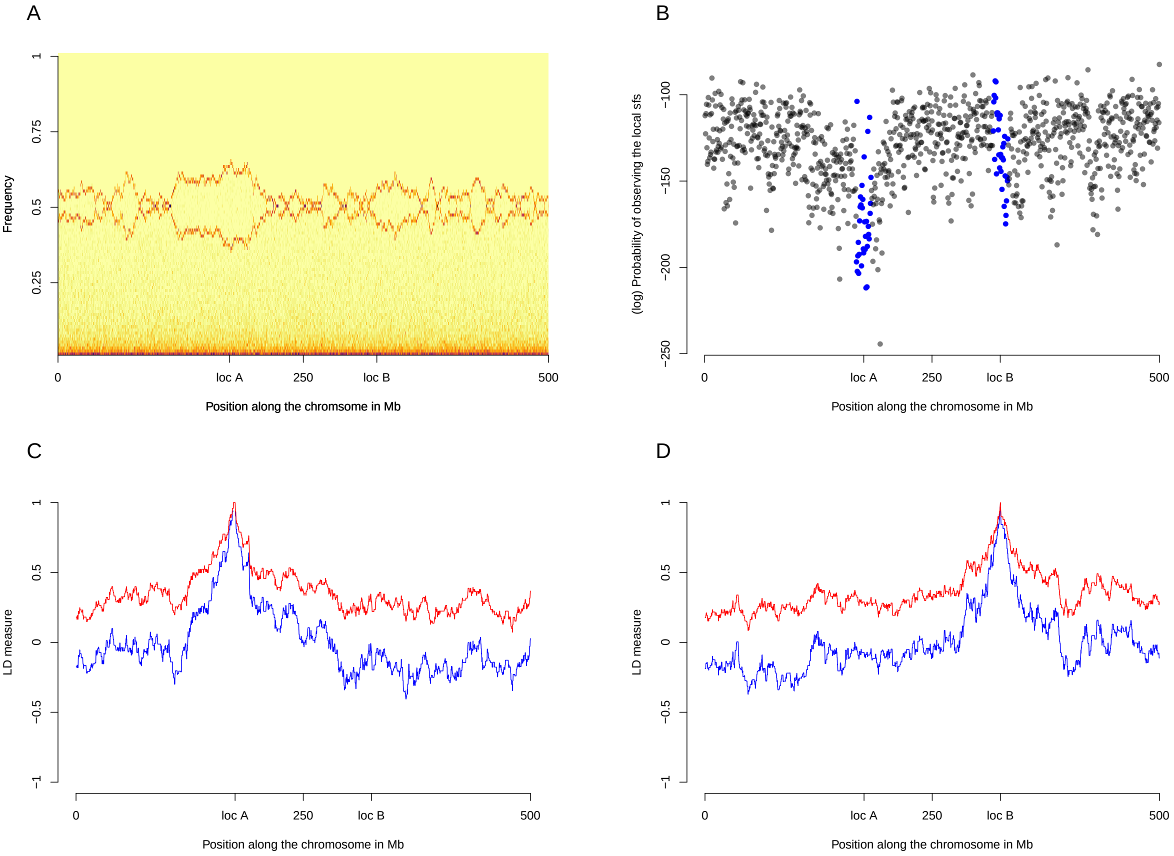

Figure S13: Illustration for the "high migration recessive" scenario. A/ Local site frequency spectrum calculated over regions of 100kb. The X axis corresponds to the position along the chromosome, the Y axis to the frequency and the density is given by color, with yellow denoting a lack of SNPs with the corresponding frequency and black an abundance of them. B/ Probability of observing the local site frequency spectrum based on the global site frequency spectrum. Windows considered as "true positive" (based on Table 2, 16 on each side of A and B) are colored in blue. C (resp. D)/ Linkage disequilibrium between locus A (resp. locus B) and the whole chromosome using uniquely fixed differences between the two parental population, calculated between alleles fixed in the same population (in red, the correlation coefficient, and in blue, the partial correlation given the genome-wide ancestry proportion). The LD between alleles A and B has therefore the opposite sign and is given by $r_{AB} = -0.289$ for the correlation coefficient and $r_{|a,AB} = 0.0715$ for the partial correlation given the genome-wide ancestry proportion. Parameters used: $s = 0, \epsilon = -0.1, m = 0.05, gen. = 1,000$

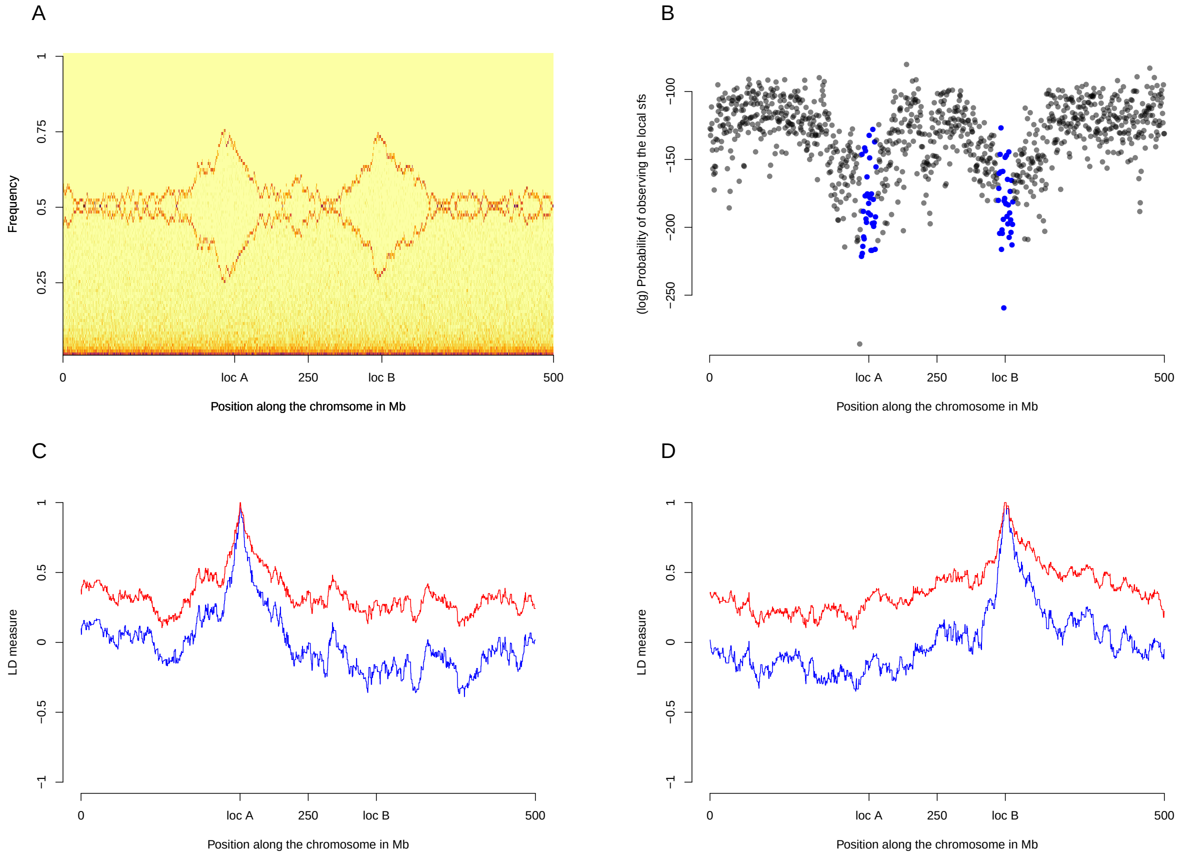

Figure S14: Illustration for the “high migration high epistatic codominant selection” scenario. A/ Local site frequency spectrum calculated over regions of 100kb. The X axis corresponds to the position along the chromosome, the Y axis to the frequency and the density is given by color, with yellow denoting a lack of SNPs with the corresponding frequency and black an abundance of them. B/ Probability of observing the local site frequency spectrum based on the global site frequency spectrum. Windows considered as “true positive” (based on Table 2, 16 on each side of A and B) are colored in blue. C (resp. D)/ Linkage disequilibrium between locus A (resp. locus B) and the whole chromosome using uniquely fixed differences between the two parental population, calculated between alleles fixed in the same population (in red, the correlation coefficient, and in blue, the partial correlation given the genome-wide ancestry proportion). The LD between alleles A and B has therefore the opposite sign and is given by $r_{AB} = -0.3$ for the correlation coefficient and $r_{|a,AB} = 0.174$ for the partial correlation given the genome-wide ancestry proportion. Parameters used: $s = 0, \epsilon = -0.5, m = 0.05, gen. = 1,000$

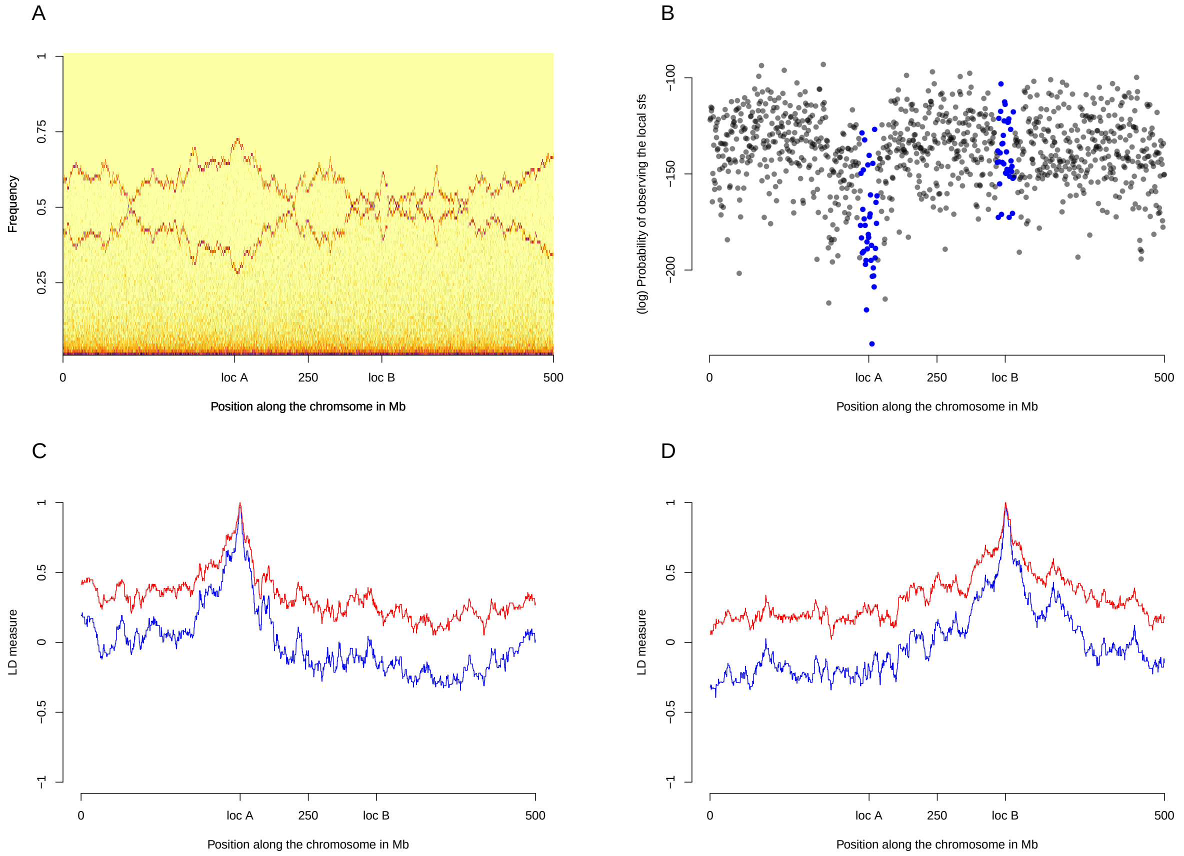

Figure S15: Illustration for the “high migration high epistatic recessive selection” scenario. A/ Local site frequency spectrum calculated over regions of 100kb. The X axis corresponds to the position along the chromosome, the Y axis to the frequency and the density is given by color, with yellow denoting a lack of SNPs with the corresponding frequency and black an abundance of them. B/ Probability of observing the local site frequency spectrum based on the global site frequency spectrum. Windows considered as “true positive” (based on Table 2, 16 on each side of A and B) are colored in blue. C (resp. D)/ Linkage disequilibrium between locus A (resp. locus B) and the whole chromosome using uniquely fixed differences between the two parental population, calculated between alleles fixed in the same population (in red, the correlation coefficient, and in blue, the partial correlation given the genome-wide ancestry proportion). The LD between alleles A and B has therefore the opposite sign and is given by $r_{AB} = -0.255$ for the correlation coefficient and $r_{|a,AB} = 0.175$ for the partial correlation given the genome-wide ancestry proportion. Parameters used: $s = 0, \epsilon = -0.5, m = 0.05, gen. = 1,000$

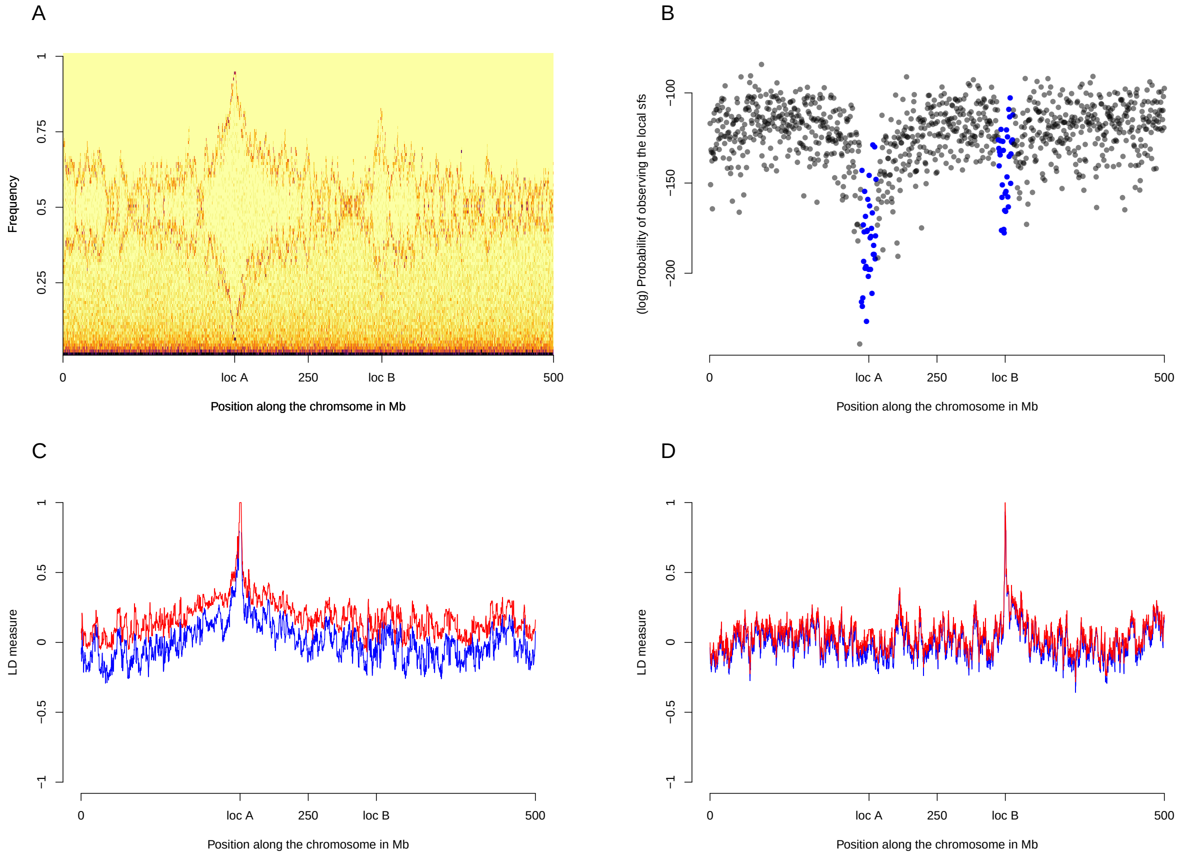

Figure S16: Illustration for the "single locus and epistatic selection" scenario. A/ Local site frequency spectrum calculated over regions of 100kb. The X axis corresponds to the position along the chromosome, the Y axis to the frequency and the density is given by color, with yellow denoting a lack of SNPs with the corresponding frequency and black an abundance of them. B/ Probability of observing the local site frequency spectrum based on the global site frequency spectrum. Windows considered as "true positive" (based on Table 2, 16 on each side of A and B) are colored in blue. C (resp. D)/ Linkage disequilibrium between locus A (resp. locus B) and the whole chromosome using uniquely fixed differences between the two parental population, calculated between alleles fixed in the same population (in red, the correlation coefficient, and in blue, the partial correlation given the genome-wide ancestry proportion). The LD between alleles A and B has therefore the opposite sign and is given by $r_{AB} = -0.0923$ for the correlation coefficient and $r_{|a,AB} = 0.0153$ for the partial correlation given the genome-wide ancestry proportion. Parameters used: $s = -0.02, \epsilon = -0.1, m = 0.005, gen. = 1,000$

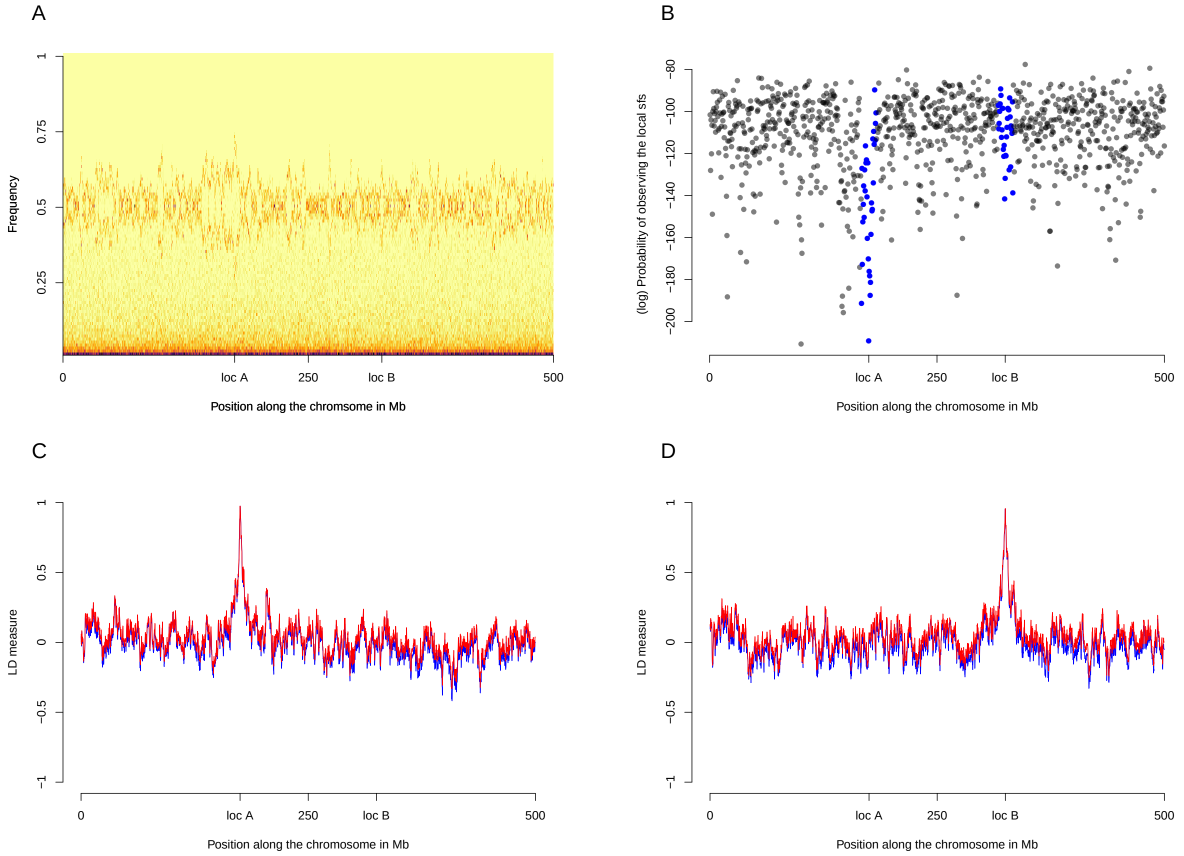

Figure S17: Illustration for the "single locus and epistatic recessive selection" scenario. A/ Local site frequency spectrum calculated over regions of 100kb. The X axis corresponds to the position along the chromosome, the Y axis to the frequency and the density is given by color, with yellow denoting a lack of SNPs with the corresponding frequency and black an abundance of them. B/ Probability of observing the local site frequency spectrum based on the global site frequency spectrum. Windows considered as "true positive" (based on Table 2, 16 on each side of A and B) are colored in blue. C (resp. D)/ Linkage disequilibrium between locus A (resp. locus B) and the whole chromosome using uniquely fixed differences between the two parental population, calculated between alleles fixed in the same population (in red, the correlation coefficient, and in blue, the partial correlation given the genome-wide ancestry proportion). The LD between alleles A and B has therefore the opposite sign and is given by $r_{AB} = -0.0526$ for the correlation coefficient and $r_{|a,AB} = -0.0258$ for the partial correlation given the genome-wide ancestry proportion. Parameters used: $s = -0.02, \epsilon = -0.1, m = 0.005, gen. = 1,000$

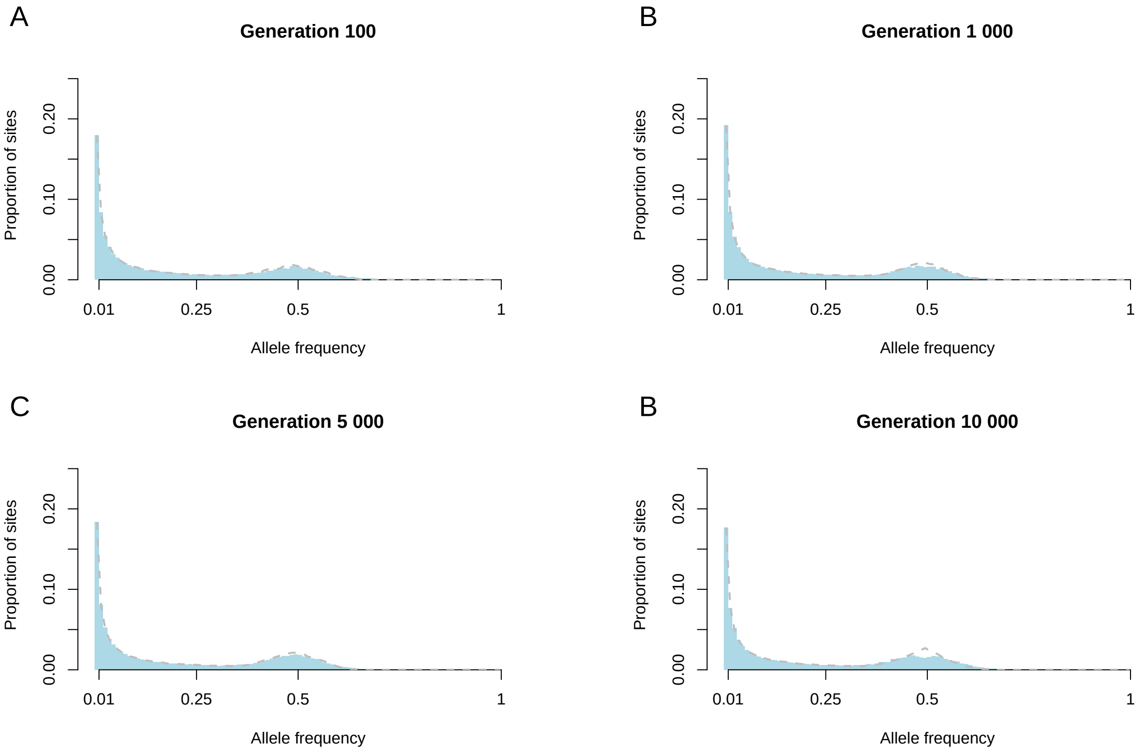

Figure S18: Genome-wide site frequency spectrum for a hybrid population under epistatic selection $\epsilon = -0.1$ for all 4 different time sample (panel A *gen.* = 100, B *gen.* = 1000, C *gen.* = 5000 and D *gen.* = 10000. The gray dashed line corresponds to the SFS of a neutral hybrid population under similar conditions.)

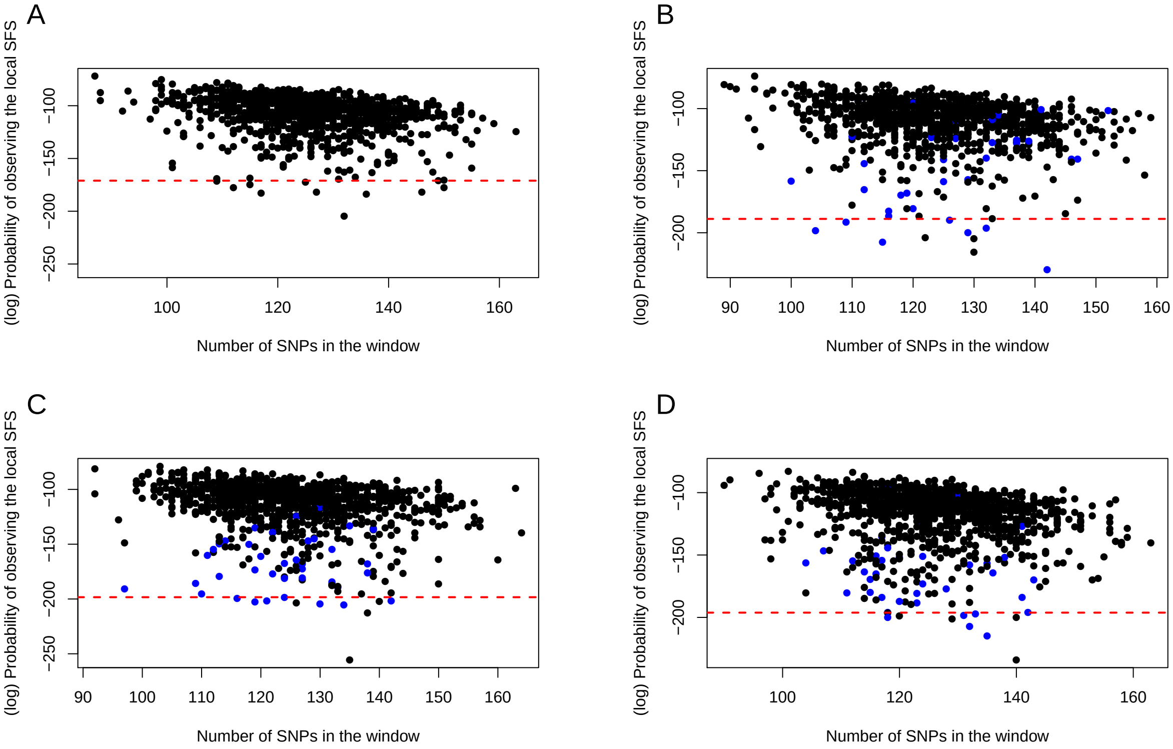

Figure S19: Probability of the local SFS based on the genome wide SFS as a function of the number of SNPs in the window used to calculate the local SFS after 1000 generations. The red line indicates the windows found in 1% quantile of the probability distribution. Windows less than 10 windows away from the selected/epistatic loci are colored in blue. A/ Neutral scenario B/ Direct selection ($s = -0.02$) C/ Epistatic selection ($e = -0.1$) D/ Strong epistatic selection ($e = -0.5$). For all cases, migration was $m = 0.005$

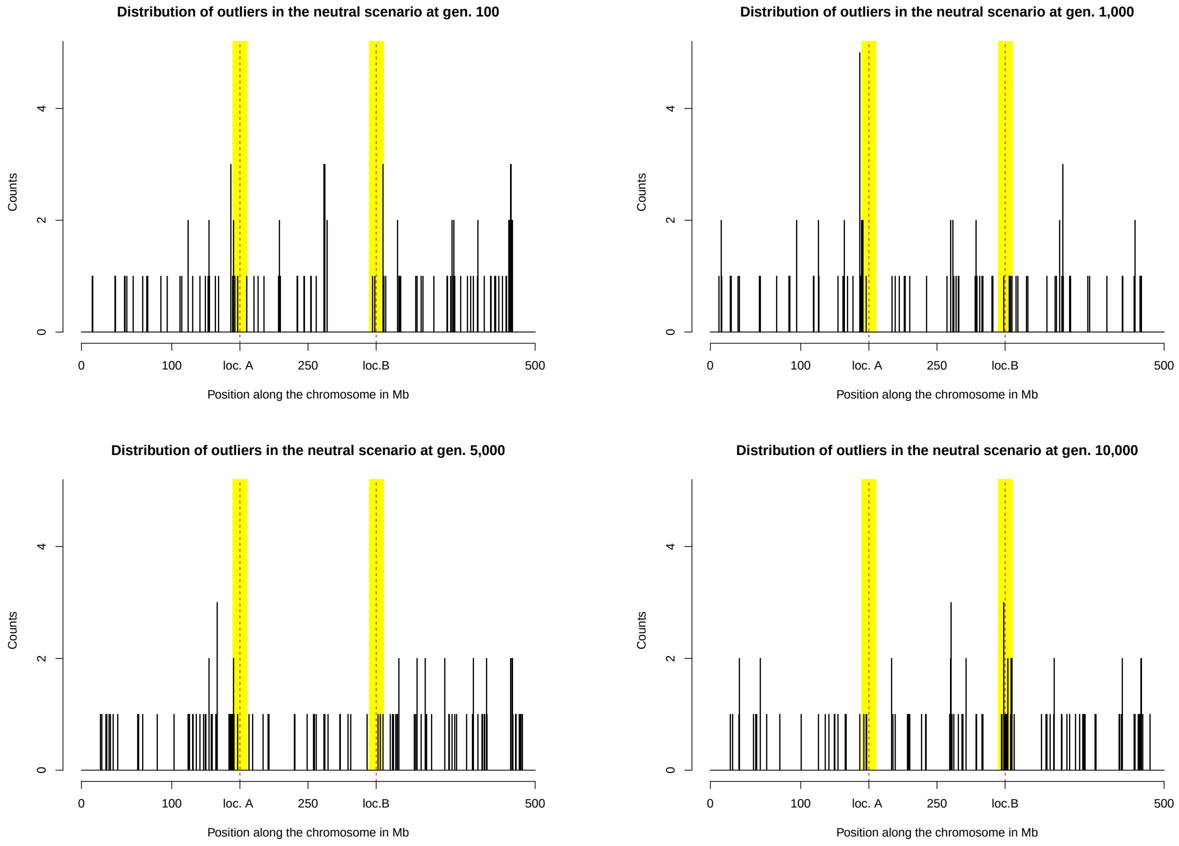

Figure S20: Distribution of outliers for the neutral scenarios. Here, outliers are defined using the following thresholds: $d = 16$, $thr_1 = 400$ and $thr_2 = 40$ and correspond to the best of combination $\{d, thr_1, thr_2\}$ (see Table S2). Red lines indicates the position of the focal loci A and B, and the yellow area corresponds to the region that are counted as true positive.

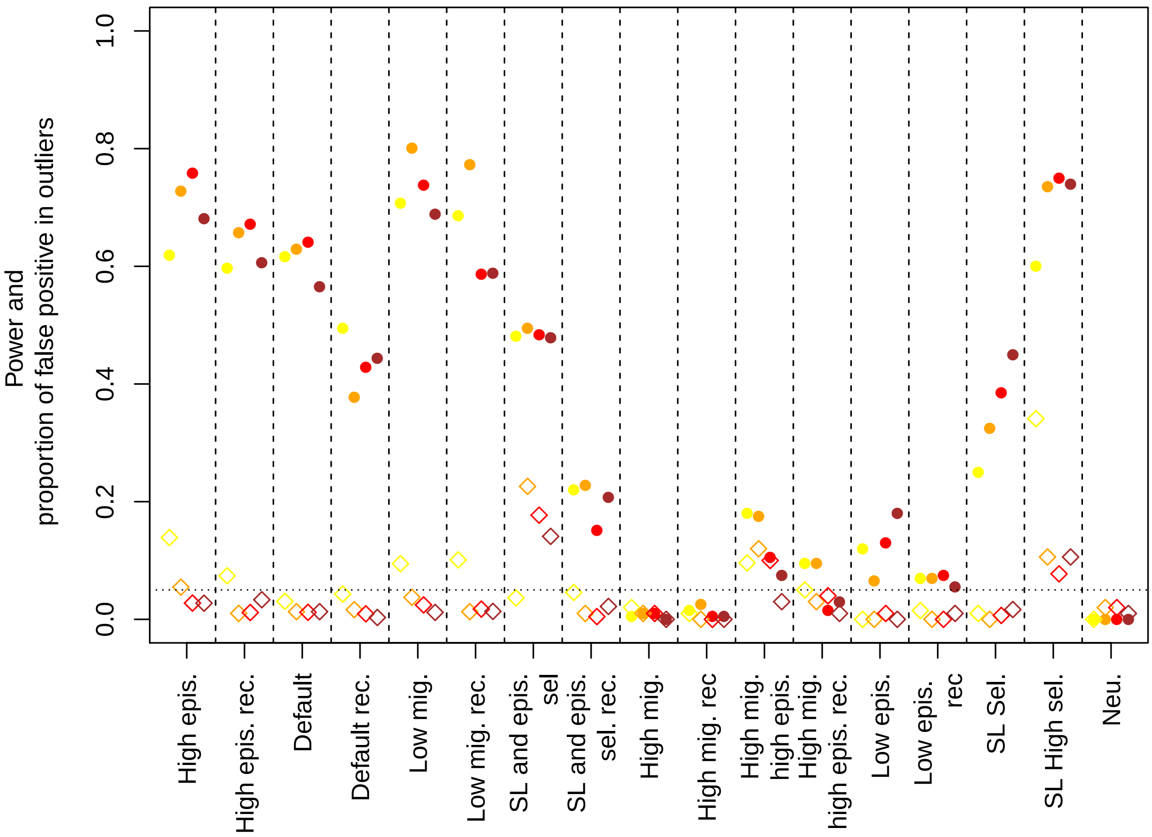

Figure S21: Power (circle) and proportion of false positives among the outliers (diamond) for the various time points (100 generations in yellow; 1 000 in orange; 5 000 in red and 10 000 in brown) using the following combination of criteria: $\{d = 9, thr_1 = 900, thr_2 = 80\}$. The values displayed correspond to the average over the various iterations, using only the complementary dataset.

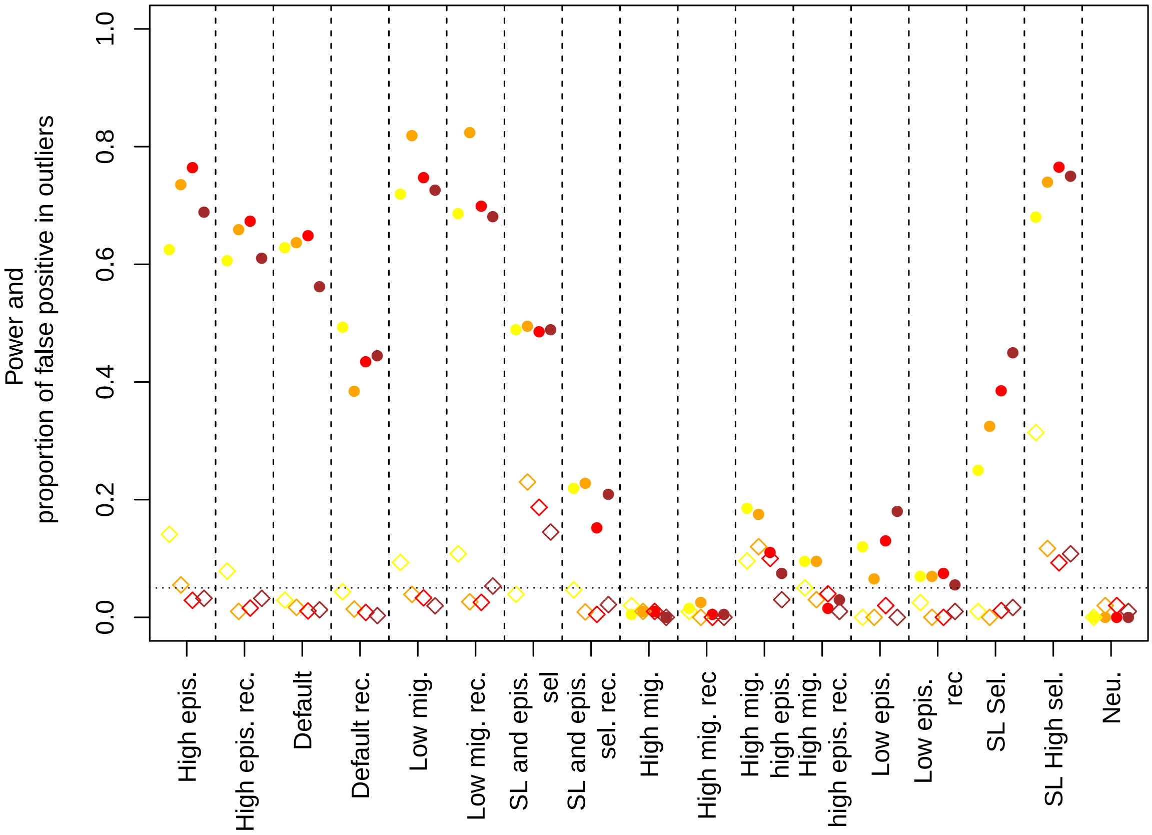

Figure S22: Power (circle) and proportion of false positives among the outliers (diamond) for the various time points (100 generations in yellow; 1 000 in orange; 5 000 in red and 10 000 in brown) using the following combination of criteria: $\{d = 9, thr_1 = 900, thr_2 = 60\}$. The values displayed correspond to the average over the various iterations, using only the complementary dataset.

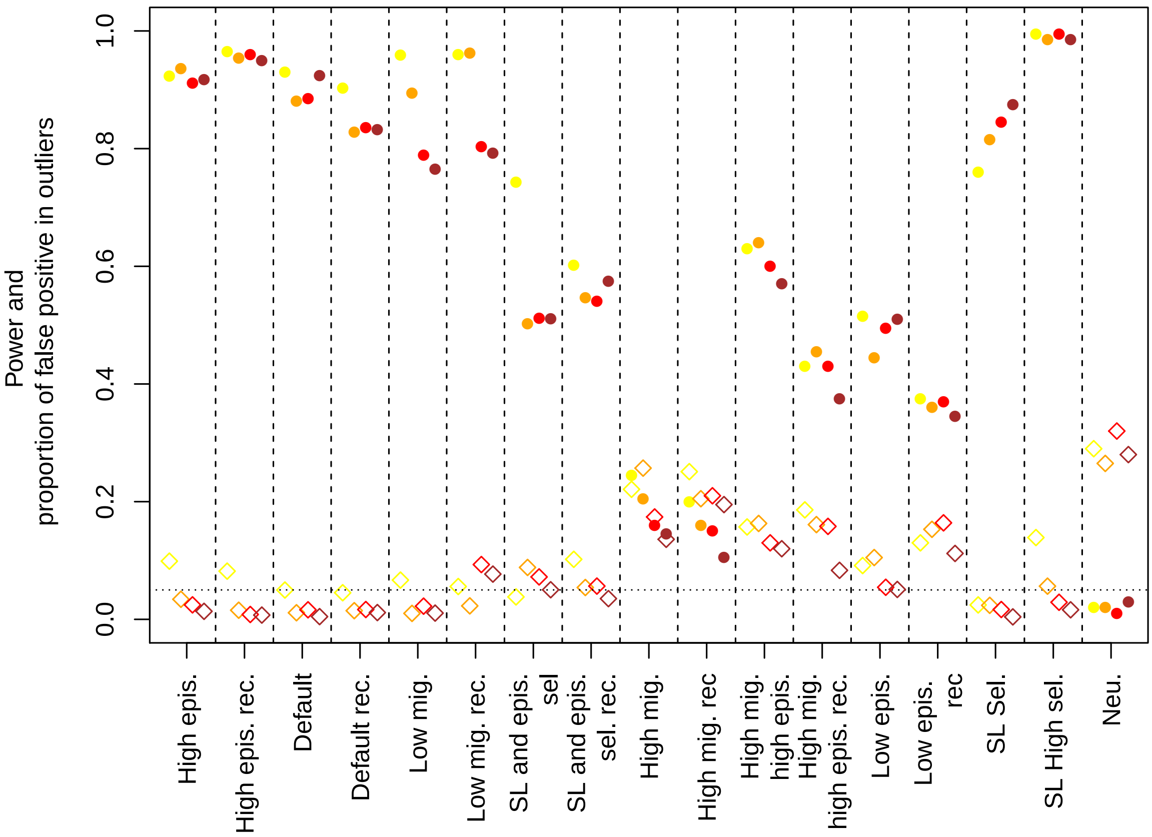

Figure S23: Power (circle) and proportion of false positives among the outliers (diamond) for the various time points (100 generations in yellow; 1 000 in orange; 5 000 in red and 10 000 in brown) using the following combination of criteria: $\{d = 16, thr_1 = 550, thr_2 = 40\}$. The values displayed correspond to the average over the various iterations, using only the complementary dataset.

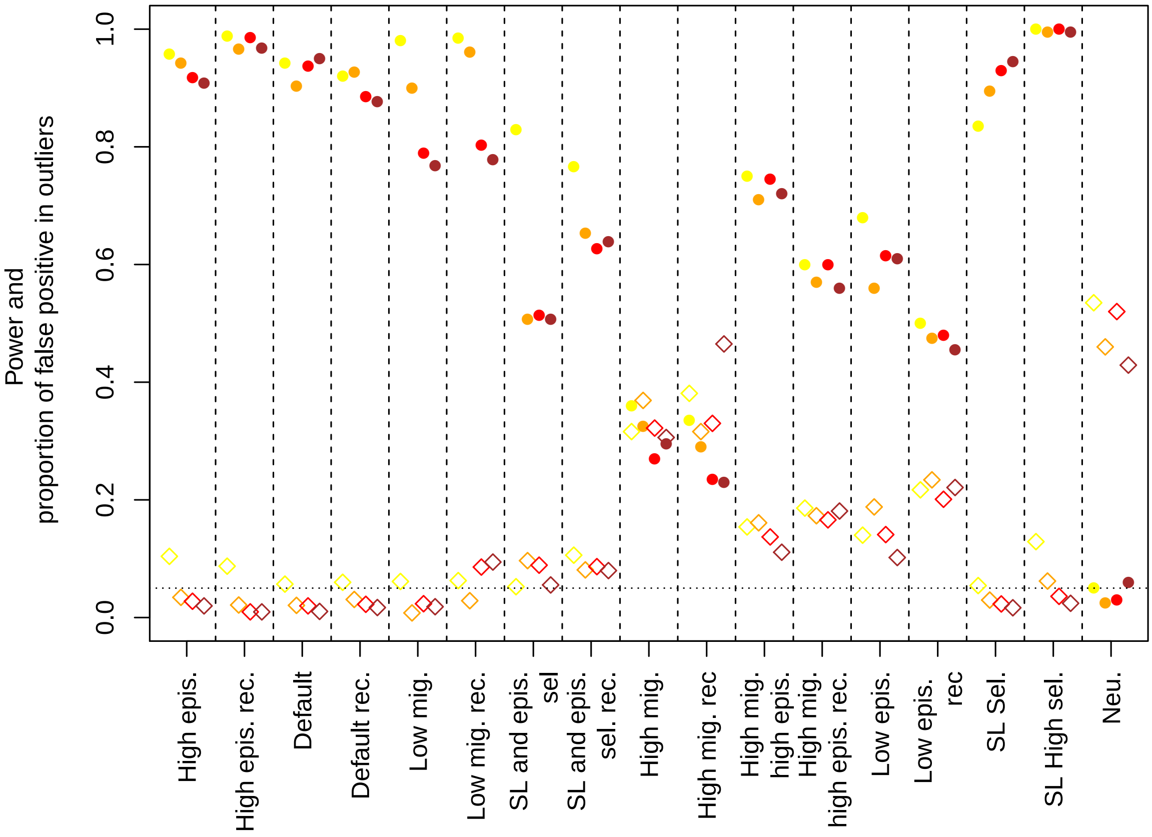

Figure S24: Power (circle) and proportion of false positives among the outliers (diamond) for the various time points (100 generations in yellow; 1 000 in orange; 5 000 in red and 10 000 in brown) using the following combination of criteria: $\{d = 16, thr_1 = 400, thr_2 = 40\}$. The values displayed correspond to the average over the various iterations, using only the complementary dataset.

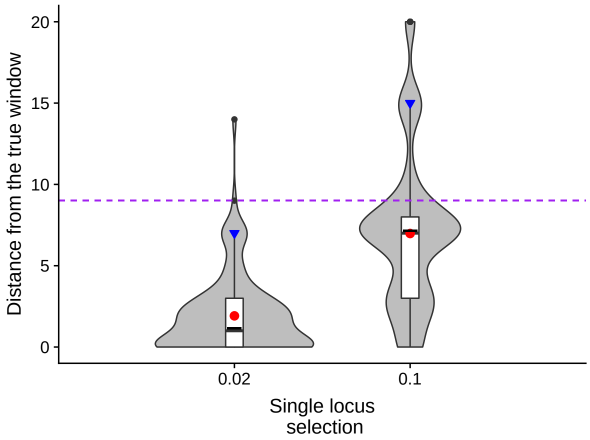

Figure S25: Distance between the detected outliers and the incompatibility loci for different strengths of the selection using $\{d = 9, thr_1 = 900, thr_2 = 80\}$ for the default migration rate m=0.005.

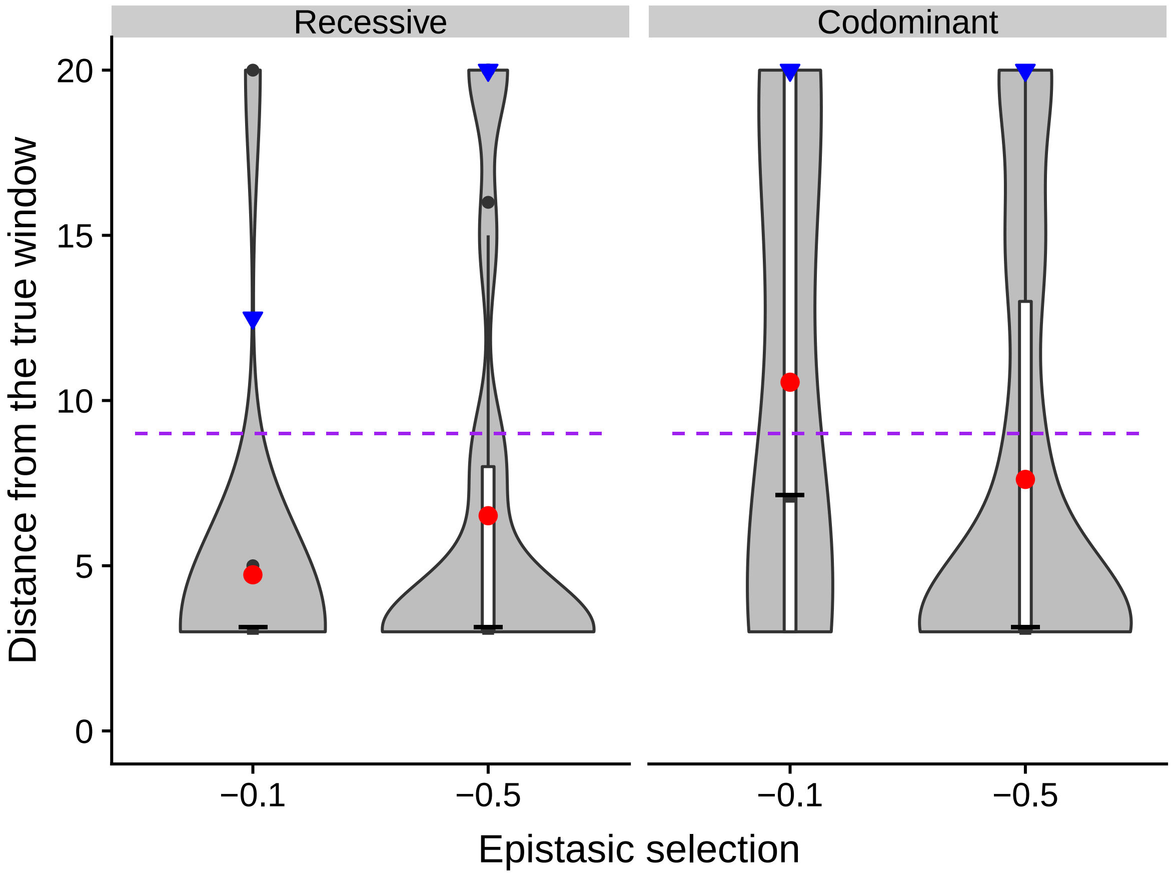

Figure S26: Distance between the detected outliers and the incompatibility loci for different strengths of the incompatibility using $\{d = 9, thr_1 = 900, thr_2 = 80\}$ for high migration rate m=0.05.

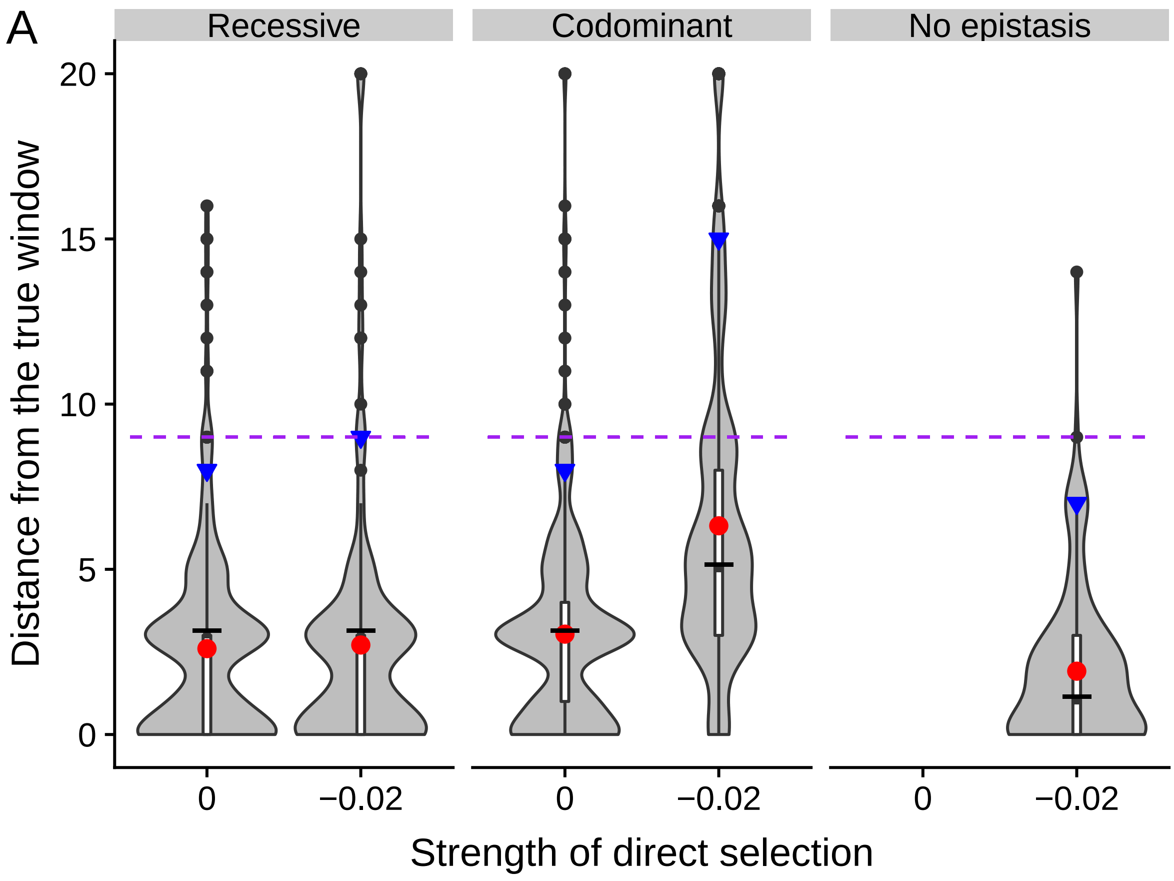

Figure S27: Distance between the detected outliers and the incompatibility loci in the absence and presence of antagonistic selection using $\{d = 9, thr_1 = 900, thr_2 = 80\}$ for epistasis $\epsilon = -0.1$. The right panel displays the distance between the detected outliers only in the presence of selection

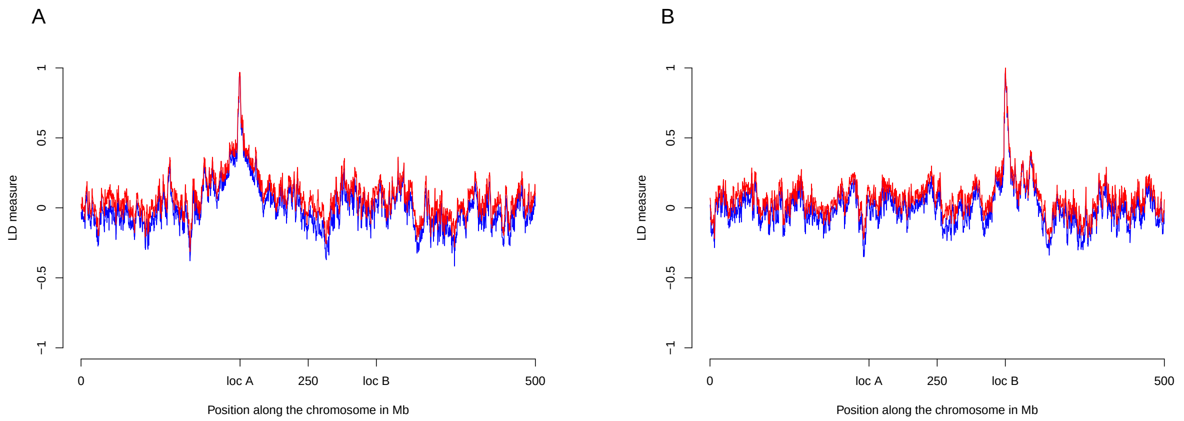

Figure S28: Illustration for the “default” scenario. A (resp. B)/ Linkage disequilibrium between locus A (resp. locus B) and the whole chromosome using uniquely fixed differences between the two parental population (in red, the correlation coefficient, $r_{AB} = -0.132$ and in blue, the partial correlation given the genome-wide ancestry proportion $r_{|a,AB} = -0.0414$) Parameters used: $s = 0, \epsilon = -0.1, m = 0.005, gen. = 1,000$

d	thr_1	thr_2	Count (best)	Count (top5%)
16	400	40	61	61
16	450	40	20	87
17	400	40	6	73
18	400	40	6	94
16	500	40	4	99
18	450	40	3	100
16	550	40	0	100
17	500	40	0	100
18	500	40	0	100
19	450	40	0	100
19	500	40	0	99
19	400	40	0	97
17	550	40	0	96
17	450	40	0	86
18	550	40	0	74
16	600	40	0	43
19	550	41	0	41
15	600	40	0	26
17	600	40	0	21
15	550	40	0	20
18	600	40	0	10

Table S1: Distribution of combination of $\{d, thr_1, thr_2\}$ using *power* as the optimizing metric. Left column corresponds to the number of a time a combination is the best one, the right one to the number of time a combination is within 5% of the best combination. Only combination that appeared twice as the best combination, or 5 times within 5% of the best combination.

A/

Scenario	Prop. false pos. in outliers	Power	False pos. rate
Default	0.0262	0.938	$2.31 * 10^{-5}$
Default rec.	0.0345	0.905	$2.44 * 10^{-4}$
High ep.	0.0504	0.934	$4.67 * 10^{-4}$
High ep. rec.	0.0313	0.973	$2.88 * 10^{-4}$
Low mig.	0.0269	0.864	$2.44 * 10^{-4}$
Low mig. rec.	0.0696	0.887	$4.41 * 10^{-4}$
SL-sel and ep.	0.0736	0.59	$6.89 * 10^{-4}$
SL-sel and ep. rec.	0.088	0.667	$3.9 * 10^{-4}$

B/

Scenario	Prop. false pos. in outliers	Power	False pos. rate
Neutral	0.486	0.0412	$1.04 * 10^{-3}$
SL-sel.	0.0284	0.882	$1.61 * 10^{-4}$
High SL-sel.	0.0602	0.996	$5.01 * 10^{-4}$
Low ep.	0.143	0.616	$4.71 * 10^{-4}$
Low ep. rec.	0.218	0.478	$6.13 * 10^{-4}$
High mig.	0.328	0.312	$9.02 * 10^{-4}$
High mig. rec.	0.373	0.272	$9.10 * 10^{-4}$
High mig high sel.	0.141	0.731	$8.65 * 10^{-4}$
High mig high sel. rec.	0.176	0.582	$8.65 * 10^{-4}$

Table S2: Ability to detect the focal loci using the "optimal" combination of criteria: $\{d = 16, thr_1 = 400, thr_2 = 40\}$, as determined by the cross-validation analysis for the various scenarios. Here, the correction for multiple testing is a factor 4/100, meaning that the false positive rate should be below $2 * 10^{-3}$. Table A displays scenarios used during the cross-validations and therefore corresponds to the average over the 61 cases where $\{16, 400, 40\}$ was the best combination or within a 5% distance of it. Table B corresponds to the other scenarios.
